# Lysine demethylase KDM1A promotes cell growth *via* FKBP8–BCL2 axis in hepatocellular carcinoma

**DOI:** 10.1016/j.jbc.2022.102374

**Published:** 2022-08-13

**Authors:** Suli Lv, Xuefeng Zhao, Erlei Zhang, Yingying Yan, Xianyun Ma, Neng Li, Qingli Zou, Lidong Sun, Tanjing Song

**Affiliations:** 1 Department of Biochemistry and Molecular Biology, School of Basic Medicine, Tongji Medical College, Huazhong University of Science and Technology, Wuhan, China; 2 Hepatic Surgery Center, Tongji Hospital, Tongji Medical College Huazhong University of Science and Technology, Wuhan, China; 3 Key Laboratory of Organ Transplantation, Chinese Academy of Medical Sciences, Wuhan, China; 4 Cell Architecture Research Institute, Huazhong University of Science and Technology, Wuhan, Hubei, China

**Keywords:** KDM1A, FKBP8, BCL2, demethylation, acetylation, KAT8, liver cancer, BCL2, B-cell lymphoma-2, CaM, calmodulin, CCK-8, Cell Counting Kit-8, CDS, coding sequence, FKBP8, FKBP prolyl isomerase 8, GST, glutathione-*S*-transferase, HA, hemagglutinin, HCC, hepatocellular carcinoma, HRP, horseradish peroxidase, IP, immunoprecipitation, JADE2, Jade family PHD finger 2, K117, lysine 117, KAT8, lysine acetyltransferase 8, KD, knockdown, KDM1A, lysine demethylase 1A, MS, mass spectrometry, NIM, nicotinamide, NLS, nuclear localization signal, SMYD3, SET and MYND domain–containing 3, TCGA, The Cancer Genome Atlas, TSA, trichostatin A, WB, Western blot

## Abstract

Advanced hepatocellular carcinoma (HCC) has a dismal prognosis. KDM1A (lysine demethylase 1A), overexpressed in multiple cancer types, is a lysine demethylase that targets both histone and nonhistone proteins. However, it is unclear how KDM1A expression affects HCC etiology. Here, we show that KDM1A can interact with and demethylate FKBP8 (FKBP prolyl isomerase 8), a cytoplasmic protein that regulates cell survival through the antiapoptotic protein BCL2 (B-cell lymphoma-2). We show that demethylation of FKBP8 enhances its ability to stabilize BCL2. Consistently, we observed positive correlation between KDM1A and BCL2 protein levels in liver cancer patients. Functionally, we reveal that FKBP8 demethylation by KDM1A is critical for liver cancer cell growth *in vitro* and *in vivo*. We went on to explore the mechanisms that might regulate KDM1A cytoplasmic localization. We found that the cytoplasmic localization and protein stability of KDM1A were promoted by acetylation at lysine-117 by the acetyl transferase KAT8 (lysine acetyltransferase 8). In agreement with this, we show that KDM1A–K117 (lysine 117) acetylation promotes demethylation of FKBP8 and level of BCL2. Finally, it has been shown that the efficacy of sorafenib, a first-line treatment for advanced HCC, is limited by clinical resistance. We show that KDM1A and BCL2 protein levels are increased during acquired sorafenib resistance, whereas inhibiting KDM1A can antagonize sorafenib resistance. Collectively, these results define a functional KDM1A–FKBP8–BCL2 axis in HCC.

Hepatocellular carcinoma (HCC) is one of the major causes to cancer-related mortality, with over 800,000 new cases and mortalities each year (1). Advanced HCC has dismal prognosis with half-survival time at only about 1 year. HCC is notoriously refractory to most conventional chemotherapy. Sorafenib has been a first-line target therapy for advanced HCC (2, 3). Sorafenib works as a multitarget kinase inhibitor, which blocks important pathways including Raf (the rapidly accelerated fibrosarcoma)/mitogen-activated protein kinase, c-Kit, vascular endothelial growth factor receptor and platelet-derived growth factor receptor, leading to decreased tumor angiogenesis, tumor cell proliferation, and increased tumor cell apoptosis (4). Yet, efficacy of sorafenib is limited by clinical resistance because of primary or acquired mechanisms, such as activation of epidermal growth factor receptor, PI3K/AKT, hypoxia-inducible factors, stress-coping mechanisms, and increased cancer stem cell populations (4, 5). Multiple epigenetic machineries also contribute to sorafenib resistance, including noncoding RNAs, DNA methylation, and histone modifications (5, 6). Investigation into HCC biology holds promise for new therapeutic strategies (7). KDM1A (lysine demethylase 1A), also known as LSD1 (lysine-specific histone demethylase 1), is the first identified lysine demethylase (8), which can regulate gene expression through demethylating histone (8, 9). KDM1A can also function through demethylation of nonhistones or function independent of its enzymatic activity (10, 11). KDM1A plays pleiotropic roles in physiology and pathophysiology (12, 13). It is overexpressed in multiple cancers and contributes to cancer malignancy (12, 13), including liver cancer (6, 14, 15). Previous studies showed that inhibiting KDM1A could decrease cell survival through p53 (tumor protein p53) methylation (16) and changes in gene transcription (17, 18, 19). Whether other mechanisms are involved remains elusive. KDM1A mainly localizes to the cell nucleus. N terminus of KDM1A, dispensable for its catalytic activity (20), harbors a nuclear localization signal (NLS), deletion of which causes translocation of KDM1A to cytoplasm (21). Besides, aberrant localization of KDM1A to cytoplasm is associated with pathology (22). Yet, it is currently unknown how the translocalization of KDM1A to cytoplasm and its function thereof are regulated.

FKBP8 (FKBP prolyl isomerase 8), a member of the proline-isomerase family, plays a role in immunoregulation, cellular autophagy, and apoptosis (23, 24, 25). FKBP8 also regulates apoptosis in different cancer types (26, 27). FKBP8 promotes resistance to apoptosis in cancers of epithelial origin (26, 28). Antiapoptotic role of FKBP8 is also supported by *in vivo* study with genetically modified mouse models (29, 30). Mechanistic studies reveal that FKBP8 regulates apoptosis through regulating the stability and localization of BCL2 (B-cell lymphoma-2) (26, 27). The effect on BCL2 relies on the interaction of FKBP8 with and activation by Ca^2+^/calmodulin (CaM) (28, 31). Thus, FKBP8 is considered as an important contributor to cellular resistance to chemotherapy (32). Yet, how function of FKBP8–BCL2 axis is regulated in HCC is not clear.

In this study, we found that KDM1A could be acetylated at lysine-117 in the NLS by lysine acetyltransferase 8 (KAT8), increasing cytoplasmic level of KDM1A. Cytoplasmic KDM1A could then interact with and demethylate FKBP8, which enhanced its ability to stabilize BCL2 in HCC cells. We further found that KDM1A–BCl2 axis was increased during acquired resistance to sorafenib, and inhibiting this signal axis could overcome sorafenib resistance.

## Results

### Cytoplasmic KDM1A promotes HCC cell growth

**Figure 1:**
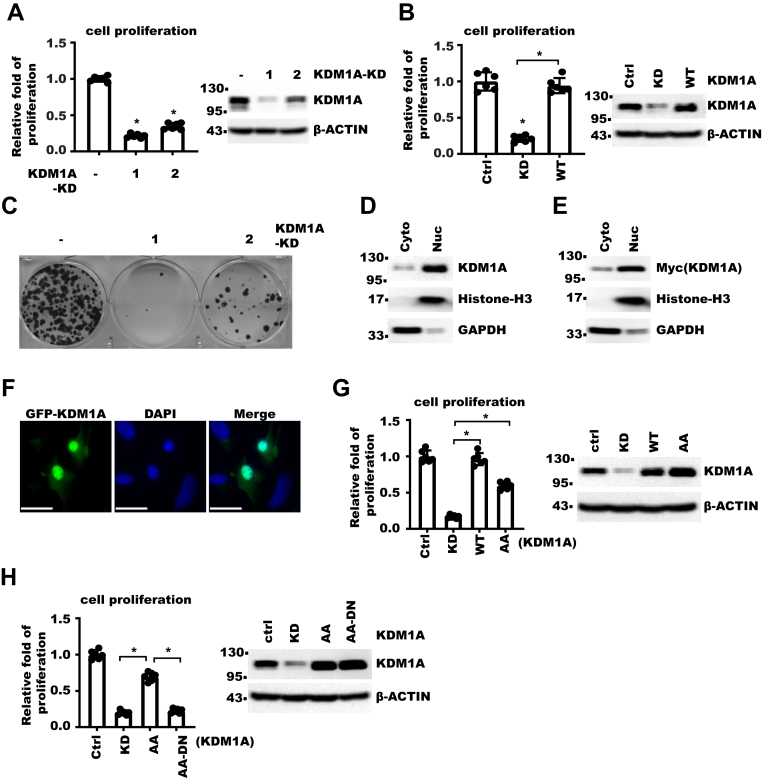
**Cytoplasmic KDM1A promotes HCC cell growth**. *A* and *B*, *left panel*, same number of HLF control or KDM1A-KD cells were seeded. Cell proliferation over 5 days was measured with CCK-8. Fold of growth was normalized to that in control cells. *Right panel* shows Western blot (WB) for indicated cells. Error bars denote standard deviation of six biological replicates. *p* Value was calculated by one-way ANOVA followed by pair-wise comparison as indicated. *C*, 500 HLF control or KDM1A-KD cells were seeded in 6-well plate. About 14 days later, cell colonies were stained with *crystal violet*. *D* and *E*, HLF control cells (*D*) or cells expressing Myc-KDM1A (*E*) were fractionated into cytoplasm (Cyto) and nucleus (Nuc). Shown are the WB results with indicated antibodies. *F*, GFP-KDM1A were expressed in HLF cells with lentivirus. Shown are photos taken with fluorescent microscopy. The scale bar represents 20 μm. *G* and *H*, *left panel*, same number of HLF KDM1A-KD and rescue cells were seeded. Cell proliferation over 5 days was measured with CCK-8. Fold of growth was normalized to that in control cells. *Right panel* shows WB for indicated cells. (Ctrl for control, KD for KDM1A-KD, WT for KDM1A-WT rescue, AA for K114A/R115A rescue, AA-DN for K114A/R115A–A539E/K661A rescue). Error bars denote standard deviation of six biological replicates. *p* Value was calculated by one-way ANOVA. CCK-8, Cell Counting Kit-8; HCC, hepatocellular carcinoma; KD, knockdown; KDM1A, lysine demethylase 1A.

Analysis of proteomic data from 159 pairs of HCC tumor and normal tissues (33) showed that KDM1A protein level was increased in cancer compared with normal tissue (Fig. S1*A*). Survival analysis of the same dataset (Fig. S1*B*) and The Cancer Genome Atlas (TCGA) dataset (Fig. S1*C*) showed that higher KDM1A level correlated with poorer prognosis in HCC patients. To examine the role of KDM1A in HCC cell growth, we knocked down KDM1A in HLF human liver cancer cells and detected significant decrease in cell proliferation (Figs. 1*A* and S1*D*), which could be ameliorated by expressing exogenous KDM1A (Figs. 1*B* and S1*E*). Knocking down KDM1A decreased cell clonogenicity as well (Fig. 1*C*). Consistently, KDM1A inhibitor treatment also decreased cell proliferation significantly (Fig. S1*F*). These results showed that KDM1A was required for HCC cell proliferation. To explore potential connection between function of KDM1A and its subcellular localization, we examined localization of KDM1A. A small yet significant portion of KDM1A was detected in the cytoplasm by both subcellular fractionation and cell imaging (Fig. 1, *D*, *E*,and *F*). We next examined whether cytoplasmic KDM1A might affect cell proliferation. In KDM1A-KD (knockdown) cell, we rescue-expressed KDM1A–K114A/R115A mutant, which predominantly localized to the cytoplasm (Fig. S1, *G* and *H*) as reported (21). We found that KDM1A–K114A/R115A (referred to as KDM1A-AA hereafter) significantly rescued defect in cell proliferation (Figs. 1*G* and S1*I*) in enzymatic activity–dependent manner (Figs. 1*H* and S1*J*). In conclusion, these data showed that KDM1A partially localized to cytoplasm, and cytoplasmic KDM1A could promote HCC cell growth.

### FKBP8 interacts with KDM1A and mediates cytoplasmic KDM1A function

**Figure 2:**
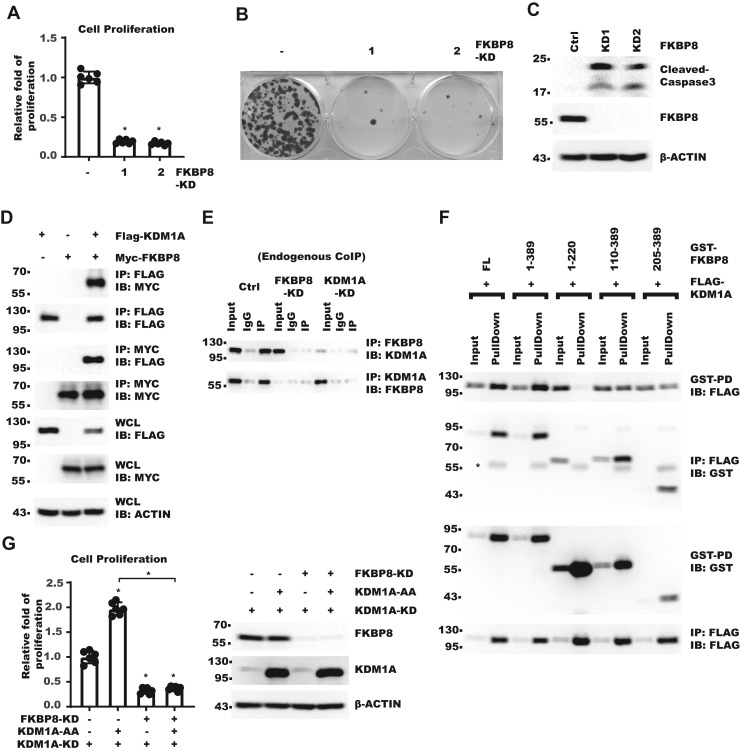
**FKBP8 interacts with KDM1A and mediates cytoplasmic KDM1A function**. *A*, same number of HLF-control or FKBP8-KD cells were seeded. Cell proliferation over 5 days was measured with CCK-8. Fold of growth was normalized to that in control cells. Error bars denote standard deviation of six biological replicates. *p* Value was calculated with one-way ANOVA followed by pair-wise comparison as indicated. *B*, 500 HLF-control or FKBP8-KD cells were seeded. About 14 days later, cell colonies were stained with crystal violet. *C*, shown are Western blot (WB) of HLF control and FKBP8-KD cell lysates. *D*, Myc-FKBP8 and FLAG-KDM1A were cotransfected into 293T cells. Shown are results of co-IP–WB. *E*, Co-IP–WB for endogenous FKBP8 and KDM1A in HLF cells. *F*, GST-FKBP8 fragments and FLAG-KDM1A were cotransfected into 293T cells. Shown are results of WB for samples from co-IP and GST-pulldown. *Asterisk* denotes band from immunoglobulin G (IgG) heavy chain. *G*, HLF KDM1A-KD cells were treated as indicated. *Left*, same number of indicated HLF cells were seeded. Cell proliferation over 5 days was measured with CCK-8. Fold of growth was normalized to that in control cells. Error bars denote standard deviation of six biological replicates. *Right*, WB result for the whole cell lysates. *p* Value was calculated with one-way ANOVA followed by pair-wise comparison as indicated. CCK-8, Cell Counting Kit-8; co-IP, coimmunoprecipitation; FKBP8, FKBP prolyl isomerase 8; GST, glutathione-*S*-transferase; KD, knockdown; KDM1A, lysine demethylase 1A.

To explore the mechanism underlying regulation of cell proliferation by cytoplasmic KDM1A, we performed mass spectrometry (MS) to identify the interactome of KDM1A (Table S2). Interestingly, FKBP8 was identified, which was reported to localize to cytoplasm and regulate apoptosis. Immunostaining of myc-FKBP8 in AD293 cell and HLF confirmed that FKBP8 mainly localized to the cytoplasm (Fig. S2, *A* and *B*). Proteomic study (33) showed that FKBP8 protein level was also increased in HCC tissue compared with normal liver (Fig. S2*C*). Meanwhile, TCGA dataset suggested that higher FKBP8 level correlates with poorer survival in liver cancer patients (Fig. S2*D*). To examine the role of FKBP8 in HCC, we knocked down FKBP8 in HLF and detected significant decrease in cell growth and clonogenesis (Figs. 2*A,* S2*E* and 2*B*) accompanied with apoptosis (Fig. 2*C*). Next, we confirmed the MS result with coimmunoprecipitation (IP) and found that both exogenous and endogenous FKBP8 interacted with KDM1A (Figs. 2*D*, S2*F* and 2*E*). Domain mapping showed that the C-terminal part of FKBP8 cytoplasmic domain mediated interaction with KDM1A (Figs. 2*F* and S2*G*). Importantly, while KDM1A-AA increased cell proliferation in KDM1A-KD cell, such effect was largely abolished by FKBP8-KD (Figs. 2*G* and S2*H*). Collectively, these data showed that KDM1A interacted with FKBP8 and indicated FKBP8 was a critical cytoplasmic effector of KDM1A.

### FKBP8 is demethylated by KDM1A

**Figure 3:**
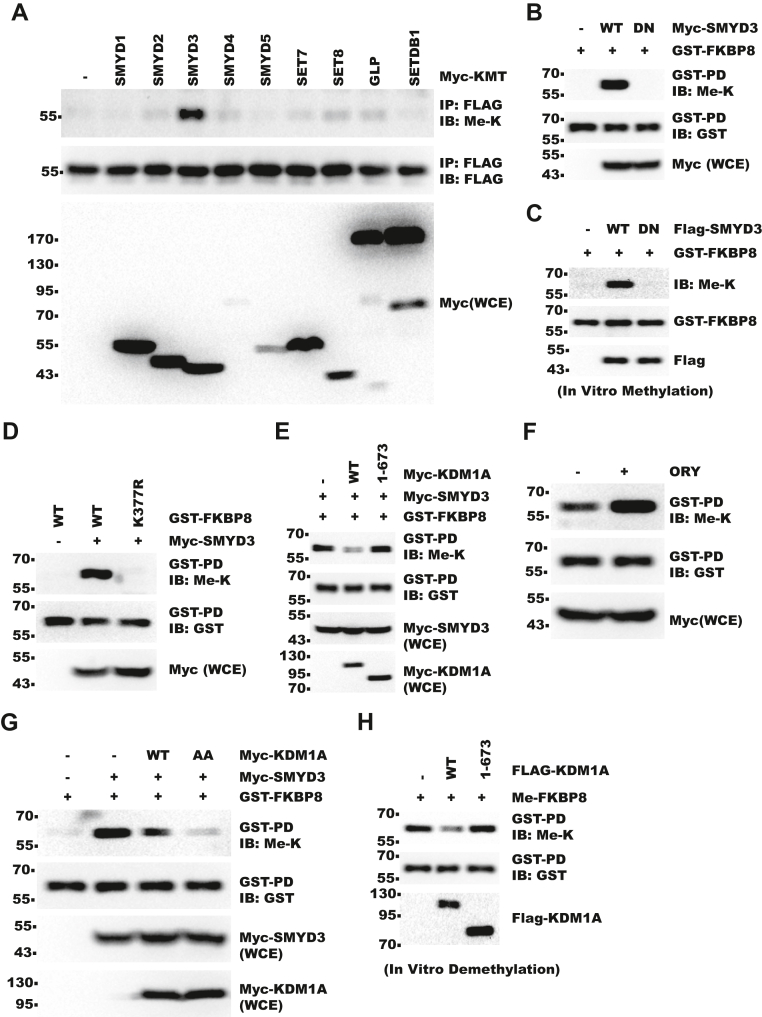
**FKBP8 is demethylated by KDM1A.***A*, FLAG-FKBP8 was cotransfected with Myc-tag methyltransferases into 293T cells. Methylation of FKBP8 was analyzed with IP–WB. (Me-K means methyl-lysine). *B*, GST-FKBP8 (110–389) was cotransfected with Myc-SMYD3-WT or Myc-SMYD3-DN (Y239F inactive mutant) into 293T cells. Shown are results of WB for GST-pulldown (PD) samples. *C*, recombinant GST-FKBP8 (110–389) was *in vitro* methylated with immunopurified FLAG-SMYD3. Reactant was analyzed with WB. *D*, GST-FKBP8 (110–389), -WT, or -K377R was cotransfected with Myc-SMYD3 into 293T cells. Shown are results of WB for GST-PD samples. *E*, GST-FKBP8 (110–389), Myc-SMYD3, and Myc-KDM1A were cotransfected into 293T cells. Shown are results of WB for GST-PD samples. *F*, GST-FKBP8 (110–389) was coexpressed with Myc-SMYD3 in 293T cells. Cells were then treated with 10 μM ORY1001 for 48 h before harvest. Shown are results of IP–WB. *G*, GST-FKBP8 (110–389) was coexpressed with Myc-SMYD3 and Myc-KDM1A in 293T cells. Cells were analyzed with IP–WB as indicated. *H*, methylated GST-FKBP8 (110–389) was affinity purified from 293T cells cotransfected with GST-FKBP8 and Myc-SMYD3. GST-FKBP8 was then demethylated *in vitro* by immunopurified FLAG-KDM1A. Reactant was analyzed with WB. FKBP8, FKBP prolyl isomerase 8; GST, glutathione-*S*-transferase; IP, immunoprecipitation; KDM1A, lysine demethylase 1A; SMYD3, SET and MYND domain–containing 3; WB, Western blot.

As the ability of KDM1A to promote cell growth in cytosol was dependent on its enzymatic activity (Fig. 1), we next explored whether FKBP8 was subject to lysine methylation. We tested potential activity of a panel of methyltransferases on FKBP8 and found that SMYD3 (SET and MYND domain–containing 3) increased methylation of FKBP8 (Fig. 3*A*). Consistently, SMYD3 was partially localized to cytoplasm in both AD293 and HLF cells (Fig. S3, *A* and *B*). Only SMYD3 WT but not catalytically inactive mutant could increase FKBP8 methylation (Fig. 3*B*) in a dosage-dependent manner (Fig. S3*C*). With *in vitro* methylation assay, we showed that SMYD3 directly methylated FKBP8 (Fig. 3*C*). Next, we set out to identify the methylated site. With deletion mutants, we narrowed down the methylation site to region 365 to 388 amino acids (Fig. S3*D*). Consistently, MS identified K377 to be methylated (Fig. S3*E*). Indeed, mutation of K377 but not adjacent lysines of FKBP8 to arginine abolished its methylation by SMYD3 (Figs. 3*D* and S3*F*). Next, we examined whether KDM1A could demethylate FKBP8. Coexpression with KDM1A-WT but not inactive mutant decreased FKBP8 methylation (Fig. 3*E*).Consistently, treatment with KDM1A selective inhibitor ORY1001 (TargetMol; catalog no.: T6922) increased FKBP8 methylation (Fig. 3*F*). In addition, KDM1A-AA demethylated FKBP8 more effectively than its WT counterpart, consistent with the cytoplasmic localization of FKBP8 (Fig. 3*G*). We then performed *in vitro* demethylation assay and further confirmed that KDM1A directly demethylated FKBP8 (Fig. 3*H*). Collectively, these data showed that KDM1A demethylated FKBP8–K377 in the cytoplasm.

### KDM1A regulates BCL2 protein level *via* FKBP8 demethylation

**Figure 4:**
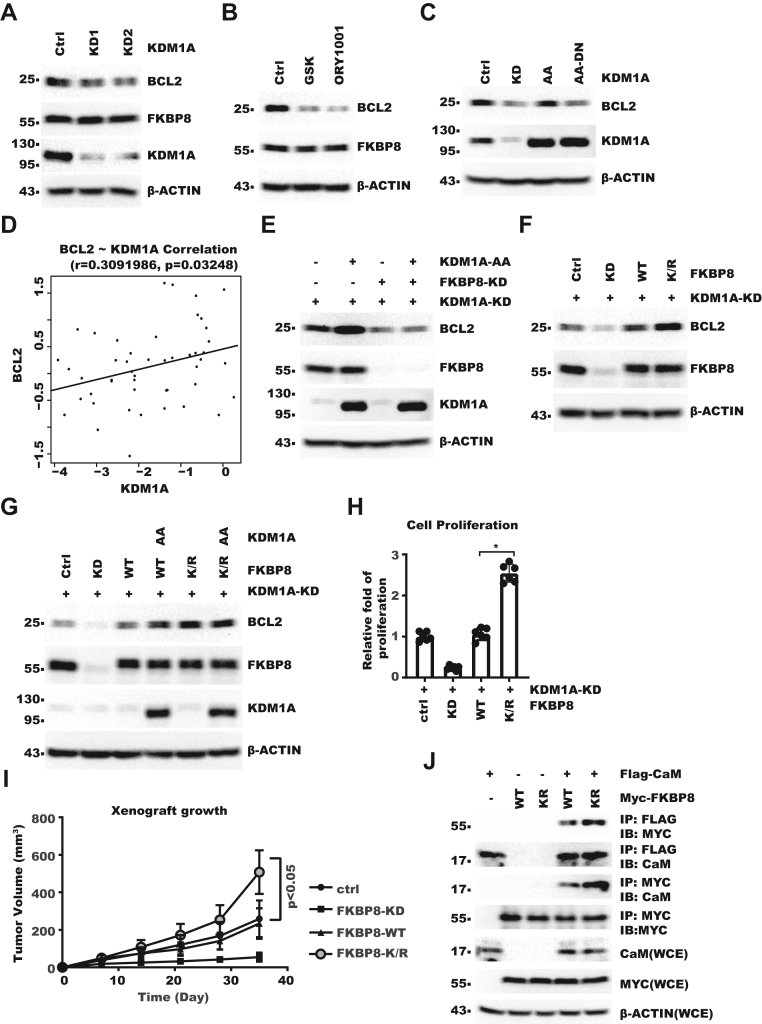
**KDM1A regulates BCL2 protein level *via* FKBP8 demethylation**. *A*, HLF control or KDM1A-KD cells were analyzed with WB. *B*, HLF cells were treated with 10 μM GSK2879552 (GSK) or 10 μM ORY1001 for 5 days. Cell lysates were analyzed with WB. *C*, HLF KDM1A-KD and rescue cells were analyzed with WB. *D*, scatter plot of KDM1A and BCL2 protein levels in liver cancer patient samples. Protein levels were calculated as log2(density/density of GAPDH). Density of each lane was normalized to HLF on the same blot. *E*, HLF KDM1A-KD, FKBP8-KD, and rescue cells were analyzed with WB. *F*, FKBP8 was knocked down in KDM1A-KD HLF cells and then FKBP8-WT or FKBP8–K377R was rescue expressed. Cell lysates were analyzed with WB. *G*, FKBP8-WT or FKBP8–K377R and/or KDM1A-AA were expressed in KDM1A-KD HLF cells. Cell lysates were analyzed with WB. *H*, FKBP8 was knocked down in KDM1A-KD HLF cells and then FKBP8-WT or FKBP8–K377R was rescue expressed. Same number of cells were then seeded. Cell proliferation over 5 days were measured with CCK-8. Fold of growth was normalized to that in control cells. Error bars denote standard deviation of six biological replicates. *p* Value was calculated with one-way ANOVA followed by pair-wise comparison as indicated. *I*, same cells as in (*H*) were injected subcutaneously into nude mice. Shown is the growth curve of xenograft. *p* < 0.05 denotes the comparison between FKBP8-WT and FKBP8–K/R rescue cells. *p* Value was calculated with two-way ANOVA followed by pair-wise comparison as indicated. *J*, Myc-FKBP8 was coexpressed with FLAG-CaM in 293T KDM1A-KD cells. Cells were harvested for IP–WB analysis as indicated. BCL2, B-cell lymphoma-2; CaM, calmodulin; CCK-8, Cell Counting Kit-8; FKBP8, FKBP prolyl isomerase 8; IP, immunoprecipitation; KD, knockdown; KDM1A, lysine demethylase 1A; WB, Western blot.

FKBP8 was previously reported to regulate BCL2 protein level (27). Consistently, FKBP8-KD also decreased BCL2 protein level in HLF cells (Fig. S4*A*). We then examined whether KDM1A might affect FKBP8–BCL2 axis. KDM1A knocking-down or inhibitor treatment decreased the protein level of BCL2 but not FKBP8 (Fig. 4, *A* and *B*). We found that expressing KDM1A-AA in KDM1A-KD cell restored BCL2 protein level (Fig. 4*C*) but not mRNA level (Fig. S4*B*). To corroborate the connection between KDM1A and BCL2, we analyzed the protein level of KDM1A and BCL2 in 24 pairs of HCC patient samples with Western blot (WB) (Fig. S4*C*). Besides that BCL2, FKBP8, and KDM1A were increased in tumor compared with normal tissues (Fig. S4, *C*–*F*), we also observed significant correlation between KDM1A and BCL2 protein level (Fig. 4*D*). Next, we examined whether FKBP8 was involved in regulation of BCL2 by KDM1A. Knocking down FKBP8 abolished the effect of KDM1A-AA on BCL2, suggesting that cytoplasmic KDM1A regulated BCL2 through FKBP8 (Fig. 4*E*). To examine whether KDM1A regulated BCL2 through demethylating FKBP8, we rescue-expressed FKBP8-WT or FKBP8–K377R (referred to as FKBP8–K/R hereafter) in cells with both FKBP8-KD and KDM1A-KD. The result showed FKBP8–K/R more potently restored BCL2 protein level than FKBP8-WT (Fig. 4*F*). Furthermore, while overexpressing KDM1A-AA increased BCL2 in FKBP8-WT cells, little effect was seen in FKBP8–K/R cells (Fig. 4*G*). Indeed, FKBP8–K/R was more potent than WT in promoting cell proliferation *in vitro* in multiple HCC cell lines (Figs. 4*H* and S4, *G*–*J*). We further confirmed that FKBP–K/R was more potent in promoting xenograft tumor growth in nude mice model (Fig. 4*I*). These results showed that KDM1A upregulated BCL2 through FKBP8 demethylation. We next explored how FKBP8–K377 methylation affected FKBP8 function. Regulation of BCL2 by FKBP8 was reported to depend on its interaction with CaM. We found FKBP8–K/R bound CaM more efficiently than WT, and methylation indeed reduced FKBP8 binding with CaM (Figs. 4*J* and S4*K*). Collectively, these data showed that KDM1A increased BCL2 protein level through demethylating FKBP8.

### KDM1A–lysine 117 is acetylated by KAT8

**Figure 5:**
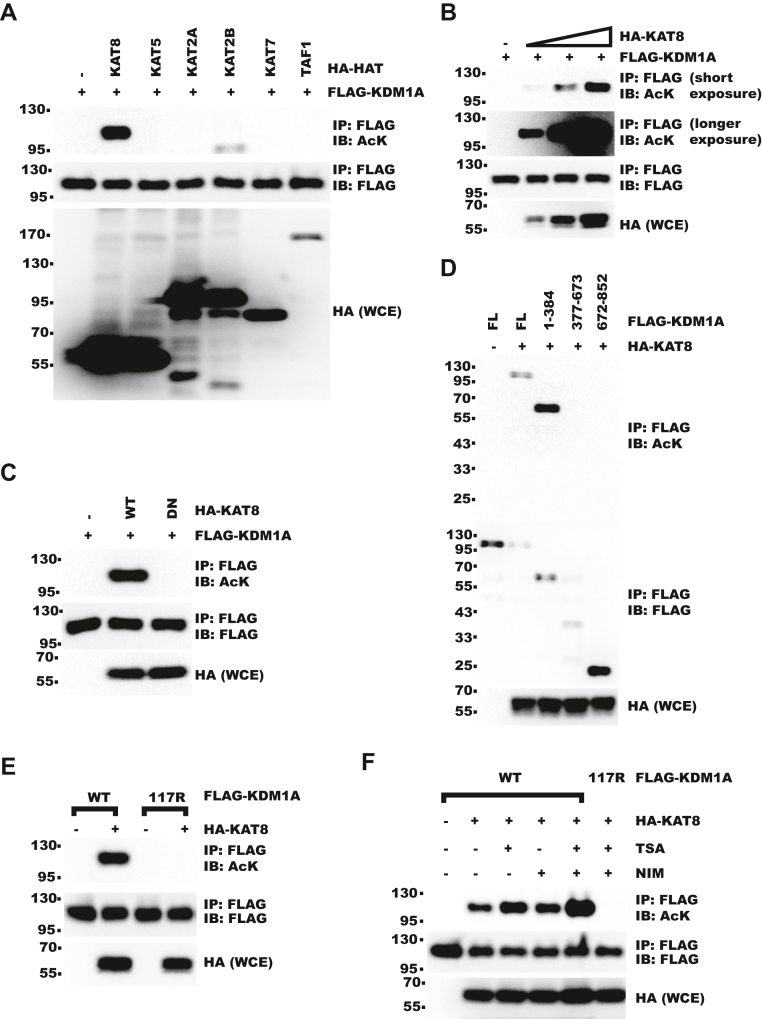
**KDM1A–K117 is acetylated by KAT8.***A*, FLAG-KDM1A was cotransfected with HA-tagged acetyltransferases into 293T cells. KDM1A acetylation was analyzed with IP–WB. *B*, FLAG-KDM1A was cotransfected with increasing HA-KAT8 into 293T cells. KDM1A acetylation was analyzed with IP–WB. *C*, FLAG-KDM1A was cotransfected with HA-KAT8-WT or inactive -K274A (DN) into 293T cells. KDM1A acetylation was analyzed with IP–WB. *D*, FLAG-KDM1A fragments were cotransfected with HA-KAT8 into 293T cells. FLAG-KDM1A acetylation was analyzed with IP–WB. *E*, FLAG-KDM1A-WT or -K117R was cotransfected with KAT8 into 293T cells. KDM1A acetylation was analyzed with IP–WB. *F*, FLAG-KDM1A-WT or -K117R was cotransfected with KAT8 into 293T cells. Cells were then treated with 3.3 μM TSA and/or 20 mM NIM for 4 h before collection. KDM1A acetylation was analyzed with IP–WB. HA, hemagglutinin; IP, immunoprecipitation; K117, lysine 117; KAT8, lysine acetyltransferase 8; KDM1A, lysine demethylase 1A; NIM, nicotinamide; TSA, trichostatin A; WB, Western blot.

As FKBP8 was regulated by cytoplasmic KDM1A (Fig. 3*G*), we next explored what might regulate KDM1A cytoplasmic localization. KDM1A was previously shown to be acetylated (34, 35). We tested the activity of a panel of histone acetyltransferases on KDM1A (Fig. 5*A*) and found that KAT8 increased acetylation of KDM1A. KAT8-WT increased KDM1A acetylation in a dosage and enzymatic activity–dependent manner (Fig. 5, *B* and *C*). Next, we tried to identify the modified site on KDM1A. With deletion constructs, we found that the acetylation signal was mainly present in the N terminus of KDM1A (Figs. 5*D* and S5*A*). MS identified the evolutionarily conserved K117 (lysine 117) as acetylated in full-length KDM1A (Fig. S5, *B* and *C*). Consistently, mutation of K117 to arginine abolished the acetylation signal by KAT8 (Fig. 5*E*). Even with histone deacetylase inhibitors, trichostatin A (TSA) and nicotinamide (NIM) treatment, K117R mutation still completely abolished the acetylation signal, further confirming K117 was acetylated by KAT8 (Fig. 5*F*). In conclusion, these data showed KDM1A–K117 could be acetylated by KAT8.

### K117 acetylation promotes cytoplasmic localization and protein stability of KDM1A

**Figure 6:**
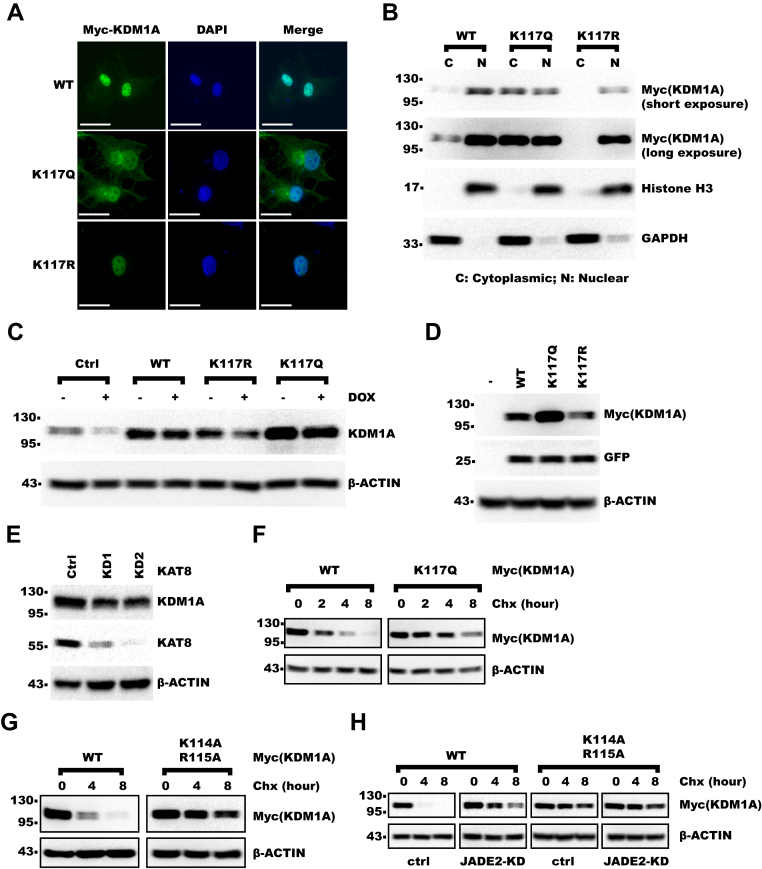
**K117 acetylation promotes cytoplasmic localization and protein stability of KDM1A.***A* and *B*, KDM1A-WT or mutants were rescue expressed in HLF KDM1A-KD cells. *A*, shown is the result of immunofluorescence with myc-tag antibody. The scale bar represents 20 μm. *B*, cells were fractionated into cytoplasmic and nuclear fractions. Fractions were analyzed with WB. *C*, KDM1A-WT or mutants were expressed in HLF cells, whereas endogenous KDM1A was knocked down with doxycycline-inducible shRNA. Cells were analyzed with WB. *D*, 0.2 μg Myc-KDM1A-WT or mutants were transiently cotransfected with 0.05 μg GFP into 293T cells in 3.5 cm dish. Cells were analyzed with WB with GFP and actin as loading control. *E*, KAT8 was knocked down in HLF cells with lentivirus-expressed shRNA. Cells were analyzed with WB. *F* and *G*, 0.4 μg Myc-KDM1A-WT or 0.2 μg -K117Q, -K114A/R115A was transfected into 293T cells in 3.5 cm dish. About 24 h later, cells were split equally into three or four dishes as indicated. The next day, cells were treated with 25 μg/ml Chx for indicated time and analyzed with WB. *H*, 0.4 μg KDM1A-WT or 0.2 μg KDM1A-K114A/K115A were transfected into control or JADE2-KD 293T cells in 3.5 cm dish. About 24 h later, cells were split equally into three dishes as indicated. The next day, cells were then treated with 25 μg/ml Chx for indicated time and analyzed with WB. Chx, cycloheximide; JADE2, Jade family PHD finger 2; K117, lysine 117; KD, knockdown; KDM1A, lysine demethylase 1A; WB, Western blot.

As K117 lied in the canonical NLS of KDM1A, we examined whether K117 acetylation might affect KDM1A localization. Indeed, mutation of K117 to glutamine mimicking lysine acetylation significantly increased KDM1A distribution in the cytoplasm as shown by both immunofluorescence (Figs. 6*A* and S6*A*) and subcellular fractionation (Figs. 6*B* and S6, *B*–*D*). In contrast, K117R mutant showed diminished localization to the cytoplasm (Fig. 6, *A* and *B*). Meanwhile, we observed that K117Q mutant was expressed at higher level than WT and K117R when expressed with same titer of virus (Fig. 6*C*). Expression of KDM1A–K117Q was also higher than KDM1A-WT and KDM1A–K117R when they were expressed in 293T cells by transient transfection (Fig. 6*D*). These results suggested that K117 acetylation could increase KDM1A protein level. Consistently, KAT8-KD decreased KDM1A protein level (Figs. 6*E* and S6*E*) but not mRNA level (Fig. S6*F*). We then treated cells with protein synthesis inhibitor, cycloheximide, for different time, and we found that KDM1A–K117Q had higher protein stability than KDM1A-WT (Fig. 6*F*). Next, we examined whether the increased protein stability could be attributed to the increased localization to the cytoplasm. Indeed, KDM1A-AA also showed higher protein stability than KDM1A-WT (Fig. 6*G*). We hypothesized that cytoplasm might provide a refuge for KDM1A from nuclear E3 ligases. Jade family PHD finger 2 (JADE2) was previously reported to be an E3 ligase of KDM1A (36). Interestingly, JADE2 mainly localized to nucleus in both HLF and 293T cells (Fig. S6, *G* and *H*). Knocking down JADE2 diminished the difference in protein stability between KDM1A-WT and KDM1A-AA (Figs. 6*H* and S6*I*). Taken together, these data showed that K117 acetylation promoted KDM1A cytoplasmic localization and protein stability.

### KDM1A–FKBP8–BCL2 axis increases cellular resistance to sorafenib

**Figure 7:**
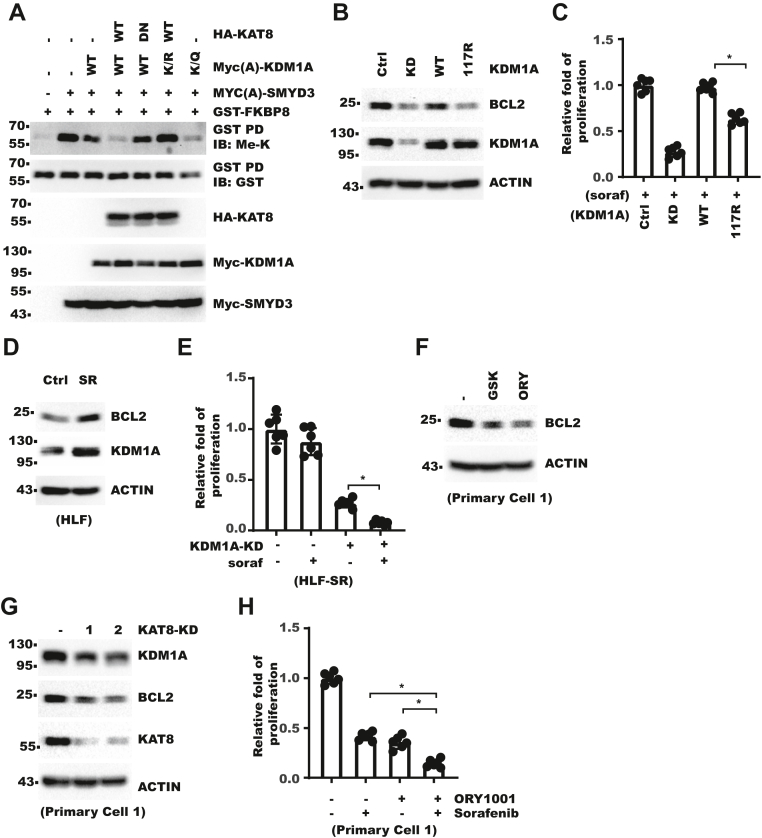
**KDM1A–FKBP8–BCL2 axis increases cellular resistance to sorafenib.***A*, 293T cells were transfected with GST-FKBP8, Myc-SMYD3, Myc-KDM1A, and HA-KAT8. Cells were analyzed with GST-pulldown (PD) and WB. *B* and *C*, KDM1A-WT or KDM1A–K117R was rescue expressed in HLF KDM1A-KD cells. Cells were analyzed with WB (*B*). In (*C*), cells were treated with 5 μM sorafenib for 80 h. Cell proliferation was measured with CCK-8. Fold of cell proliferation was normalized to that of control cells. Error bars denote standard deviation of six biological replicates. *p* Value was calculated with one-way ANOVA followed by pair-wise comparison as indicated. *D*, WB analysis for sorafenib-resistant HLF cell. *E*, KDM1A was knocked down in sorafenib-resistant HLF cells. Cells were treated with 5 μM sorafenib for 4 days. Cell proliferation was measured with CCK-8. Fold of cell proliferation was normalized to that of control. Error bars denote standard deviation of six biological replicates. *p* Value was calculated with one-way ANOVA followed by pair-wise comparison as indicated. *F*, primary HCC cell culture 1 was treated with 10 μM GSK2879552 (GSK) or 10 μM ORY1001 (ORY) for 5 days. Cells were analyzed with WB. *G*, primary HCC cell culture 1 was infected with lentivirus expressing KAT8-shRNA. After selection with puromycin for 5 days, cell lysates were analyzed with WB. *H*, primary HCC cell culture 1 was treated with 5 μM sorafenib and/or 10 μM ORY1001. Cell proliferation was measured with CCK-8. Fold of cell proliferation was normalized to that of control. Error bars denote standard deviation of six biological replicates. *p* Value was calculated with one-way ANOVA followed by pair-wise comparison as indicated. BCL2, B-cell lymphoma-2; CCK-8, Cell Counting Kit-8; FKBP8, FKBP prolyl isomerase 8; GST, glutathione-*S*-transferase; HA, hemagglutinin; HCC, hepatocellular carcinoma; KAT8, lysine acetyltransferase 8; KDM1A, lysine demethylase 1A; SMYD3, SET and MYND domain–containing 3; WB, Western blot.

We next examined whether KDM1A–K117 acetylation affected FKBP8 demethylation. *In vitro* demethylation assay showed that K117 mutation did not affect KDM1A enzymatic activity *per se* (Fig. S7*A*). However, *in vivo* demethylation of FKBP8 was promoted by K117Q mutation or coexpression with KAT8 while inhibited by K117R mutation (Fig. 7*A*), which confirmed demethylation of FKBP8 mainly occur in the cytoplasm. Consistently, K114A/R115A also showed enhanced demethylation than K117R (Fig. S7*B*). We showed that FKBP8–K377 demethylation promoted BCL2 level (Fig. 4). Consistent with its dwarfed effect on FKBP8 methylation, KDM1A–K117R was less efficient in restoring BCL2 protein level (Fig. 7*B*). As BCL2 and cellular apoptosis played a central role in HCC cell response to sorafenib (37), we next explored potential role of KDM1A–FKBP8–BCL2 axis in sorafenib response and resistance, which was clinically relevant. In control cells, KDM1A inhibition increased the efficacy of sorafenib (Fig. S7*C*). However, K117R is less potent than WT in restoring survival in sorafenib (Figs. 7*C* and S7*D*). We then established sorafenib-resistant HLF cells and found that they expressed higher levels of BCL2 and KDM1A than parental cells (Fig. 7*D*). Importantly, KDM1A inhibition restored sensitivity to sorafenib in sorafenib-resistant cells (Figs. 7*E* and S7*E*). Next, we examined whether the KDM1A–BCL2 axis also functioned in HCC patient samples. We established two primary cultures from fresh HCC patient samples. Treatment with KDM1A inhibitor decreased BCL2 in these cultures (Figs. 7*F* and S7*F*). Meanwhile, knocking down KAT8 also decreased KDM1A in these cultures (Figs. 7*G* and S7*G*). Furthermore, KDM1A inhibitor had synergistic effect with sorafenib on the proliferation of primary cells (Figs. 7*H*, and S7, *H* and *I*). Collectively, these data showed that KDM1A acetylation was critical for regulation of FKBP8–BCL2 axis by KDM1A, and targeting KDM1A–FKBP8–BCl2 axis could increase cell sensitivity to sorafenib.

## Discussion

### KDM1A–FKBP8–BCL2 axis

**Figure 8:**
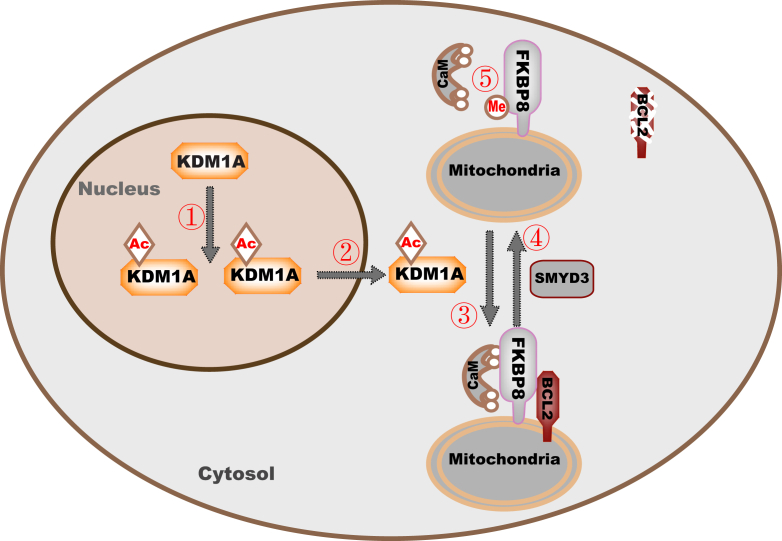
**Working model.** KDM1A is acetylated by KAT8, which increases KDM1A protein stability (①) and cytoplasmic translocation (②). In cytoplasm, KDM1A demethylates FKBP8, which promotes FKBP8 interaction with CaM and stabilizes BCL2 (③). In contrast, SMYD3 methylates FKBP8 (④), which interferes with FKBP8–CaM binding (⑤). BCL2, B-cell lymphoma-2; CaM, calmodulin; FKBP8, FKBP prolyl isomerase 8; KAT8, lysine acetyltransferase 8; KDM1A, lysine demethylase 1A; SMYD3, SET and MYND domain–containing 3.

KDM1A plays versatile roles in physiology and pathophysiology including cancer. In this study, we uncover a mechanism by which KDM1A regulates growth and sensitivity to sorafenib in HCC cancer cells. We reveal KDM1A demethylates FKBP8 in the cytoplasm, which increases BCL2 protein level, as the otherwise-methylated FKBP8 binds CaM less efficiently. Consistently, we detect positive correlation between KDM1A and BCL2 protein levels in HCC patients. In addition, we report that KDM1A, FKBP8, and BCL2 are overexpressed in HCC, which correlates with poor prognosis. We also find that targeting KDM1A–FKBP8–BCL2 axis increases cellular sensitivity to sorafenib. Besides, as demethylation of FKBP8 mainly occurs in the cytoplasm, we go on to identify KDM1A localization, and protein stability in the cytoplasm is increased by KAT8-mediated K117 acetylation (Fig. 8).

### KDM1A is regulated by K117 acetylation

While the canonical functions of KDM1A have been mainly attributed to its activity in the nucleus, recent studies and ours have shown that KDM1A may function *via* cytoplasmic substrates (38, 39). Although aberrant localization of KDM1A to cytoplasm is seen in pathology (22), little is known how KDM1A subcellular localization is regulated. In this study, we identify that acetylation of K117 can significantly increase the distribution of KDM1A into the cytoplasm. We further show that cytoplasmic KDM1A has higher protein stability because cytoplasm provides a shelter from the nuclear E3 ligase JADE2. Consistent with the role of K117 acetylation in cytoplasmic localization of KDM1A, K117R mutant has diminished cytoplasmic localization and activity on FKBP8. Interestingly, KAT8 was reported to acetylate KDM1A at sites other than K117, which could regulate transcription of KDM1A target genes (35). These data again testify to the concept that same type of modification at different residues may give different consequences.

### KDM1A inhibitor increases efficacy of sorafenib in HCC

There are huge unmet medical needs for advanced HCC patients. Poor response to chemotherapy, scarcity of available target therapy, and resistance to therapy-induced apoptosis all contribute to the dismal prognosis. Identifying novel targets or overcoming resistance to current therapy holds promise for better prognosis.

Since its approval, sorafenib has been the mainstay in advanced HCC target therapy. Even with the advent of immune checkpoint blockers, sorafenib remains an important part of HCC treatment options (40). Yet, multiple mechanisms have been uncovered to contribute to sorafenib resistance. Increasing or restoring sensitivity to sorafenib has been one of the major efforts in HCC therapy (41). BCL2 is a pivotal antiapoptotic protein, overexpression of which is associated with cancer cell sensitivity to drugs including sorafenib (37, 42). Increase in BCL2 expression was observed in cellular models of HCC sorafenib resistance, and inhibiting BCL2 diminished sorafenib resistance (37). Activity and protein stability of BCL2 is modulated by its key regulator FKBP8, in a CaM-dependent manner (28, 31). Yet whether FKBP8–BCL2 axis is dysregulated in HCC and its relation with sorafenib sensitivity was unexplored. In this study, we find that FKBP8 is overexpressed in liver cancer and correlates with poor survival in HCC patients. We show that loss of FKBP8 inhibits cell proliferation. We further find that effect of FKBP8 on BCL2 is retarded by methylation at FKBP8–K377. Conversely, demethylation of FKBP8 by KDM1A facilitates its interaction with CaM and increases BCL2 protein level. Consistently, inhibiting KDM1A inhibits HCC cell growth in synergy with sorafenib. Furthermore, we find that expression of both BCL2 and KDM1A is enhanced in cellular model of sorafenib resistance, and KDM1A inhibitor alleviates the resistance to sorafenib. In summary, in this study, we uncover a mechanism where KDM1A localization to the cytoplasm is enhanced by KAT8-mediated K117 acetylation. We further show that cytoplasmic KDM1A can increase cellular resistance to sorafenib *via* the FKBP8–BCL2 axis. Our data suggest that inhibiting KDM1A–FKBP8–BCL2 axis may benefit HCC therapy.

## Experimental procedures

### Cell culture

Human embryonic kidney 293T, AD293 (human embryonic kidney cell), HepG2, HLF, HLE, and Huh7 (human liver cancer cell) cells were obtained from Dr Shuguo Sun’s laboratory (Huazhong University of Science and Technology). Cells were cultured in Dulbecco’s modified Eagle’s medium (Procell, Inc; catalog no.: GM150210) supplemented with 10% fetal bovine serum (Gibco; catalog no.: 141-044). Cycloheximide (Sigma; catalog no.: C7698, 25 μg/ml), GSK2879552 (TargetMol; catalog no.: T4418, 10 μM), ORY1001 (10 μM), sorafenib (Alladin; catalog no.: S125098, 5 μM), TSA (MCE; catalog no.: HY-15144, 3.3 μM), NIM (Sigma; catalog no.: 72340, 20 mM), or doxycycline hyclate (MCE; catalog no.: HY-N0565B, 1 μg/ml) were added to cell culture, respectively, where indicated.

### Bacterial strains

DH5α (Tsingke Biological Technology, Inc; catalog no.: TSV-A07), Stbl3 (Shanghai Weidi Biotechnology Co; catalog no.: DL1046S), and BL21 CodonPlus (DE3)-RIPL competent cells (Shanghai Weidi Biotechnology Co; catalog no.: EC1007S) were as described previously (43).

### Patient samples

About 24 pairs of primary HCC and paired tumor-adjacent samples were collected at Tongji Hospital, Huazhong University of Science and Technology from the Department of Surgery. Apparent normal tissue was separated from cancer tissues, and necrotic tissue was discarded. Samples were frozen in liquid nitrogen until further analysis. For WB, samples were lysed in 1× Laemmli buffer with grinding and sonication. Study was approved by Medical Ethics Committee of Tongji Medical College, Huazhong University of Science and Technology and abided by the Declaration of Helsinki principles.

### WB

WB was performed as previously reported (43). The following primary antibodies were used in WB: anti-KDM1A (CST; catalog no.: 2184), anti-Histone H3 (abcam; catalog no.: 1791), anti-GAPDH (abclonal; catalog no.: AC033), anti-FKBP8 (Proteintech; catalog no.: 11173-1-AP), anti–methyl-lysine (abcam; catalog no.: ab9045), anti-BCL2 (CST; catalog no.: 15071), anti-β-actin (abclonal; catalog no.: AC026), anti–acetyl-lysine (CST; catalog no.: 9441), anti-GFP (Proteintech; catalog no.: 50430-2-AP), anti-KAT8 (CST; catalog no.: 46862), anti-JADE2 (Proteintech; catalog no.: 11513-1-AP), anti-Myc tag (Proteintech, catalog no.: 16286-1-AP; Santa Cruz, catalog no.: sc-40), antihemagglutinin (HA) tag (Covance; catalog no.: MMS-101P), horseradish peroxidase (HRP)–conjugated anti–glutathione-*S*-transferase (GST) tag (Proteintech; catalog no.: HRP-66001), anti-GST tag (Origene; catalog no.: TA150101), and HRP-anti-FLAG tag (Sigma; catalog no.: A8592). HRP-conjugated secondary antibodies used were Abclonal (catalog nos.: AS014, AS003, AD064, AS061, and AS062).

### Immunofluorescence and GFP fluorescence

For immunofluorescence, cells were fixed with 4% paraformaldehyde for 15 min at room temperature. Cells were then permeabilized with 0.5% Triton X-100 in cold PBS. After blocking with 1% bovine serum albumin, cells were incubated with primary antibodies: anti-HA tag (CST; catalog no.: 3724) and anti-myc tag (Santa Cruz; catalog no.: sc-40). Afterward, cells were washed with PBS + 0.2% Tween-20 and incubated with Alexa Fluor–conjugated secondary antibodies (Invitrogen; catalog nos.: 715-545-150, 711-585-152, and 711-545-152). Cells were then stained with 4′,6-diamidino-2-phenylindole for 5 min at room temperature. Finally, cells were washed with PBS + 0.2% Tween-20 and mounted onto a slide with mounting medium (Abcam; catalog no.: AB104135). Photos were taken with Zeiss-A1 Axiovert A1 fluorescence microscopy equipped with 63× oil objective lens with QImaging Retiga R6 Monochrome camera. GFP-expressing cells were grown on glass-bottom culture dish and treated the same as in immunofluorescence but without primary antibody or secondary antibody. All photos were processed with Fiji (NIH).

### Total RNA extraction and reverse transcription

Total RNA extraction and reverse transcription were performed as previously reported (43). Briefly, RNA was extracted with Trizol (Invitrogen; catalog no.: 15596018) following the manufacturer's instructions. DNA was removed with DNase I treatment (Sigma; catalog no.: AMPD1-1KT). Then reverse transcription was done using Improm-II reverse transcription kit (Promega; catalog no.: A3800) with random primer.

### Real-time quantitative PCR

Real-time quantitative PCR was performed as previously reported with SYBR Green Master Mix (Bio-Rad; catalog no.: 1725124) on Bio-Rad CFX connect real-time PCR machine (43). Primers are shown in Table S1.

### Establishment of sorafenib-resistant cells

Sorafenib-resistant cells were generated as previously reported (44). HLF cells were cultured with escalating concentration of sorafenib up to 7 μM during a period of 2 months. Afterward, cells were maintained with 5 μM sorafenib.

### Liver cancer cell primary culture

Primary culture was established as previously reported (45). Briefly, necrotic or apparent normal tissue was removed from patient samples. Samples were minced with scissors into 1-2 mm diameter pieces and then digested with 0.1% collagenase IV (Sigma; catalog no.: V900893) in PBS at 37 °C for 1 h with occasional gentle shaking. Cell suspension was then filtered through 100 μm Cell Strainer (Corning; catalog no.: 352360). Cells were centrifuged consecutively at 1000, 800, and 600 rpm for 5 min. Cancer cells were resuspended into YA medium containing Dulbecco’s modified Eagle’s medium, 10% fetal bovine serum, 40 ng/ml epidermal growth factor (Peprotech; catalog no.: AF-100-15), 1× insulin–transferrin–selenium (Gibco; catalog no.: 41400045), 10 μM Y-27632 (MCE; catalog no.: HY-10583), and 5 μM A83-01 (MCE; catalog no.: HY-10432A).

### Cell proliferation assay

About 1 × 10^5^ cells were seeded into 6-well plate. After cultured for several days as specified, cells were counted again with cell counter. The growth ratio in each group of cell was calculated. The growth ratios were normalized to that of control group. Sorafenib, GSK2879552, or ORY1001 was added as specified.

### Cell Counting Kit-8 assay

About 2000 cells were seeded into each well of 96-well plate. Cell proliferation was measured using Cell Counting Kit-8 (CCK-8) assay (Beyotime; catalog no.: C0038) following the manufacturer's instructions. Briefly, 10 μl CCK-8 was added to 100 μl medium. Absorbance at 450 nm was measured 1 h later.

### Lentivirus-mediated gene overexpression or KD

Lentivirus was made by cotransfecting coding sequence (CDS) (or shRNA)-expressing plasmids with pMD2.G and psPAX2 into 293T cells. About 48 h later, supernatant was filtered through 0.45 μm PES filter (Millipore; catalog no.: SLHP033RB). Virus suspension was added to cells in log phase. About 24 h later, cells were passaged and selected with antibiotics, 4 days for puromycin (InvivoGen; catalog no.: ANT-PR-1) and 7 days for neomycin (InvivoGen; catalog no.: ant-hg-5) and blasticidin (InvivoGen; catalog no.: ANT-BL-1). Sequences of shRNA are shown in Table S1. For conventional KD, pLKO-puro vector was used while for inducible KD, TET-pLKO was used (46). Cloning of shRNA-coding oligo was described as reported previously (43).

### MS

MS was performed as previously reported (43). GST-FKBP8 was cotransfected with SMYD3 into 293T cells. GST-FKBP8 was captured with GST pulldown and loaded to SDS-PAGE. Gel was stained with Coomassie blue R250. Gel band corresponding to GST-FKBP8 was cut out. To detect lysine acetylation on KDM1A by KAT8, FLAG-KDM1A was cotransfected with KAT8 into 293T cells. FLAG-KDM1A was captured with M2-agarose beads (Sigma; catalog no.: A2220) and loaded to SDS-PAGE. Gel was stained with Coomassie blue R250, and gel was then cut for further analysis. To detect KDM1A interactome, FLAG-KDM1A was captured with M2-agarose beads from transiently transfected 293T cells and loaded to SDS-PAGE. Gel was stained with Coomassie blue R250, and the whole gel lane was cut. Further procedures for MS were done by Novogene, Inc. Briefly, protein was subject to in-gel digestion with trypsin. Extracted peptides were further analyzed with LC–MS/MS.

### Transfection-acetylation/methylation assay

About 4 μg FLAG-KDM1A was cotransfected with 4 μg HA-KAT8 into 293T cells in 6-cm dish. Cells were collected into IPE150 buffer (Hepes–KOH, pH 7.5, NP-40 0.5%, NaCl 150 mM, and glycerol 10%) supplemented with 3.3 μM TSA, 20 mM NIM, and protease inhibitors. When specified, same concentrations of TSA and NIM were also added to cell culture 4 h before cell collection. After sonication, cell lysates were cleared by centrifugation. Cell lysates were then incubated with anti-FLAG M2-conjugated agarose beads. After washed with IPE1000 (Hepes—KOH, pH 7.5, NP-40 0.5%, NaCl 1000 mM, and glycerol 10%) for three times, bead-bound protein was eluded with SDS-loading buffer (1× Laemmli buffer plus 0.002% bromophenol blue) and analyzed with WB. Detection of FKBP8 *in vivo* methylation was similar except that FLAG- or GST-FKBP8 was cotransfected with Myc-SMYD3 into 293T cells. About 48 h after transfection, cells were collected without histone deacetylase inhibitors, and transfected FKBP8 was captured with M2-agarose beads (Sigma; catalog no.: A2220) or glutathione agarose beads (Sigma; catalog no.: G4510).

### Colony-formation assay

About 500 cells were seeded into 6-well plate, and medium was changed every 5 days. After 14 days, medium was discarded. Cells were washed with PBS twice and stained with crystal violet (0.5% in methanol). Plate was washed with water three times and let dry. Pictures were acquired with Epson document scanner.

### Transfection-co-IP assay

Transfection-co-IP assay was performed as previously reported (43, 47). About 48 h after transfection, cells were collected into IPE150 buffer. After sonication and centrifugation, cell lysate was incubated with anti-FLAG M2-conjugated agarose beads or myc-tag antibody. For myc-tag IP, protein-A/G-conjugated agarose beads (Transgen; catalog no.: DP501-01) were later added to cell lysate. Bead-captured material was washed with IPE150 three times and analyzed with WB.

### Endogenous co-IP assay

Endogenous co-IP assay was performed as previously reported (43). Cells were collected into IPE150 buffer. After sonication and centrifugation, cell lysate was incubated with primary antibodies (mouse anti-FKBP8; Proteintech, catalog no.: 66690-1-Ig and mouse anti-KDM1A; Zen BioScience, catalog no.: 200057). Antibody–antigen complex was captured with Protein-A/G-conjugated agarose beads and analyzed with WB.

### Pull-down assay (FLAG-CaM pulldown GST-FKBP8)

GST-FKBP8 were cotransfected with SMYD3 into 293T cells. Cells were collected into IPE150 buffer. After sonication and centrifugation, cell lysate was incubated with FLAG-CaM immunopurified with anti-FLAG M2-agarose beads from transfected cells. Beads-bound material was washed with IPE150 for three times and analyzed with WB.

### Cellular fractionation

Cells were fractionated into cytoplasm and nucleus as previously reported (43). Briefly, cells were scraped into PBS and then let swollen in buffer A (10 mM Hepes, pH 7.9, 10 mM KCl, 0.5 mM DTT, 0.5 mM PMSF, and 1.5 mM MgCl_2_) for 15 min. The cellular suspension is homogenized with a glass homogenizer with 10 up-and-down passes of the pestle. Cells were then centrifuged at 2000*g* for 1 min. The supernatant was termed cytoplasm, and the pellet was termed nucleus.

### Recombinant protein purification

GST-FKBP8 (catalog no.: 110-389) was purified following procedure reported previously (43). Briefly, BL21-codon plus competent cells were transformed with GST-FKBP8 plasmid. Single colony was picked and let grow at 37 °C to an absorbance at 600 nm between 0.6 and 0.8. IPTG was then added to culture to final concentration 0.3 mM for induction at 16 °C overnight. Bacteria were then collected by centrifugation and lysed with lysozyme treatment followed by sonication. GST-FKBP8 protein was captured by Glutathione Agarose (Sigma) and eluted with 20 mM glutathione.

### *In vitro* methylation

About 0.5 μg recombinant GST-FKBP8 was incubated with FLAG-SMYD3 immunopurified from 293T cells in histone methylation buffer (20 mM Tris–Cl, pH 8.0, and 0.5 mM DTT) supplemented with 1.2 mM S-adenosyl methionine (Sigma; catalog no.: A7007). Reaction proceeded at 30 °C for 1 h and was then stopped by adding 5× SDS-loading buffer. Reactant was analyzed with WB.

### *In vitro* demethylation

Methylated FKBP8 was affinity purified from 293T cells cotransfected with GST-FKBP8 and myc-SMYD3. FLAG-KDM1A was immunopurified from transfected 293T cells. GST-FKBP8 and FLAG-KDM1A were then incubated *in vitro* at 30 °C for 2 h in demethylation buffer (100 mM glycine, pH 8.0, and 50 mM KCl).

### Plasmid construction

Plasmid construction was performed as previously reported (43). CDS-expression plasmid was constructed by standard PCR with complementary DNA from 293T cell or Sino Biological, Inc followed by restriction digestion and ligation. shRNA plasmid was constructed with annealing each pair of oligos and ligating into vector. Plasmid sequences were validated by Sanger sequencing.

### Xenograft experiment

Experiment was done as described previously (43). All animal experiments were performed following the institute guidelines and approved by Ethics committee of Tongji Medical College and animal facility of Huazhong University of Science and Technology. The mice were acclimated to the new environment for at least 1 week. Mice were housed in ventilated cage in a temperature-controlled room (21 ± 1 °C) with a 12 h light/12 h darkness cycle. Food and water were available ad libitum. Mice were randomly assigned to experimental groups. About 5 × 10^6^ cells in 100 μl 1:1 mixture of PBS and Matrigel (BD Biosciences; catalog no.: 354248) were injected subcutaneously into flanks of 5- to 6-week-old male BALB/c nude mice (Beijing Vital River Laboratory Animal Technology). Tumor volumes were measured every week with caliper. Tumor volume was calculated as 0.5∗L∗W∗W. Before estimated tumor volume reached 1000 mm^3^, mice were euthanized.

### Reconstitution assay (rescue expression)

Exogenous KDM1A or FKBP8 was expressed in KDM1A or FKBP8 KD cells, respectively, by lentivirus-expressing shRNA-resistant CDS. shRNA resistance was generated by introducing synonymous mutation at the shRNA-target region. Specifically, “GCTACATCTTACCTTAGTCAT” to “GCCACCAGCTACTTGTCCCAC” for KDM1A-sh1383, “ACGTCGCTGGAGAATGGCACA” to “ACCAGCCTCGAAAACGGAACC” for FKBP8-sh498, and “TGAAGGTGAAGTGTCTGAACA” to “TAAAAGTCAAATGCTTAAATA” for FKBP8-sh925.

## Quantification and statistical analysis

PCR and cell proliferation results were processed with Microsoft Excel and visualized with GraphPad Prism 8.0 (GraphPad Software, Inc). Error bars in bar graphs for PCR data denote standard deviation of technical triplicates. Error bars in bar graphs of cell proliferation denote standard deviation of four biological replicates for cell counting and six biological replicates for CCK-8. GraphPad Prism 8.0 was used for statistical analysis of PCR and cell proliferation experiments. Survival information and mRNA level of KDM1A and FKBP8 for TCGA liver HCC patients are obtained from The Human Protein Atlas. Log2-transformed protein level from previous proteomic study based on MS (33) was used as is. For WB of KDM1A, FKBP8, and BCL2 in tumor and tumor-adjacent tissues, densitometry for WB bands was performed with Fiji. Protein expression levels were represented as log2(density of target/density of GAPDH). To compare the protein levels of KDM1A, FKBP8, and BCL2 in tumor *versus* tumor-adjacent tissues, *p* values were calculated with two-sided paired Student's *t* test with R basic function. Boxplots were done with “beeswarm” package and basic R in R (3.3.3) (R Core Team). Pearson correlation between KDM1A and BCL2 protein levels in patient samples was performed in Rstudio (1.0.136) (RStudio.com) with R (3.3.3) basic function. Survival analysis and Kaplan–Meier plot was done with “survival” package in R (3.3.3). Xenograft growth was analyzed with two-way ANOVA. In all cases, *p* < 0.05 was considered as significant.

## Data availability

The data that support the findings of this study are available from the corresponding authors upon reasonable request.

## Supporting information

This article contains supporting information (33).

## Conflict of interest

The authors declare that they have no conflicts of interest with the contents of this article.
