# Supplementary material for: Lysine demethylase KDM1A promotes cell growth via FKBP8–BCL2 axis in hepatocellular carcinoma
Source: J Biol Chem. 2022 Aug 13;298(9):102374. doi: 10.1016/j.jbc.2022.102374 (PMC9478407; doi:10.1016/j.jbc.2022.102374)
Supplement: Table S2 [file mmc3.pdf]

Supplementary Figure S1 related to Figure 1.
a. Boxplot for log2-transformed protein level of KDM1A in primary HCC tissues and paired normal liver tissues (data from (Gao et al., 2019)).
b. Kaplan-Meier plot for overall survival of HCC patients with high versus low KDM1A (data from (Gao et al., 2019), 79 cases low, 79 cases high).
c. Kaplan-Meier plot for survival of HCC patients with high versus low KDM1A (data from TCGA LIHC dataset, 291 cases low, 74 cases high).
d-e. Same number of HLF control or KDM1A-KD cells were seeded. Cell numbers were counted five days later. Fold of growth was normalized to that in control cells. Right panel shows western blot for indicated cells. Error bars denote standard deviation of four biological replicates. P-values (<0.05) were calculated with one-way ANOVA followed by pair-wise comparison as indicated.
f. Same number of HLF cells were seeded and treated with 10μM GSK2879552 or 10μM ORY1001. Cell numbers were counted seven days later. Fold of growth was normalized to that in control cells. Error bars denote standard deviation of four biological replicates. P-value (<0.05) was calculated with one-way ANOVA followed by pair-wise comparison as indicated.
g. Myc-KDM1A-K114A/R115A was expressed in 293T cells with transient-transfection. Cells were analyzed with immunofluorescence and nuclei were stained by DAPI. Shown are photos taken with fluorescence microscopy. Scale bar denotes 10μm.
h. Myc-KDM1A-K114A/R115A was expressed in HLF cells with lentivirus. In the left panel, cells were analyzed with immunofluorescence and nucleus was stained with DAPI. Shown are photos taken with fluorescence microscopy. Scale bar denotes 20μm. In the right panel, cells were fractionated into cytosol and nucleus and then analyzed with Western Blot.
i-j. Left panel, same number of HLF KDM1A-KD and rescue cells were seeded (AA for K114A/R115A-rescue, AA-DN for K114A/R115A-A539E/K661A-rescue). Cell numbers were counted five days later. Fold of growth was normalized to that in control cells. Error bars denote standard deviation of four biological replicates. P-values (<0.05) were calculated with one-way ANOVA followed by pair-wise comparison as indicated.

Supplementary Figure S2 related to Figure 2.
a-b. Myc-FKBP8 was expressed in AD293 (a) and HLF (b) cells with transient transfection and lentivirus transduction respectively. Cells were analyzed with immunofluorescence and nuclei were stained by DAPI. Shown are photos taken with fluorescence microscopy. Scale bars denote 20μm (a) and 10μm (b) respectively.
c. Boxplot for log2 transformed FKBP8 protein level in primary HCC tissues compared to paired normal liver tissues (data from (Gao et al., 2019)).
d. Kaplan-Meier plot for overall survival of HCC patients with high versus low FKBP8 (data from TCGA LIHC dataset, 251 cases low, 114 cases high).
e. Same number of HLF-control or FKBP8-KD cells were seeded. Cell numbers were counted five days later. Fold of growth was normalized to that in control cells. Error bars denote standard deviation of four biological replicates. P-values (<0.05) were calculated with one-way ANOVA followed by pair-wise comparison as indicated.
f. Myc-FKBP8 and FLAG-KDM1A were co-transfected into 293T cells. Shown are results of CoIP-WB.
g. Cartoon illustration of FKBP8 domains. Numbers on the top denote starting and ending positions of corresponding domains. TM stands for transmembrane domain.
h. HLF KDM1A-KD cells were treated as indicated. Left, same number of indicated HLF cells were seeded. Cell numbers were counted five days later. Fold of growth was

normalized to that in control cells. Error bars denote standard deviation of four biological replicates. P-values (<0.05) were calculated from one-way ANOVA followed by pair-wise comparison as indicated.

Supplementary Figure S3 related to Figure 3.
a-b. Myc-SMYD3 was expressed in AD293 (a) and HLF (b) cells with transient transfection and lentivirus respectively. Cells were analyzed with immunofluorescene with Myc-tag antibody. Nuclei were stained with DAPI. Shown are photos taken with fluorescence microscopy. Scale bar=20μm.
c. GST-FKBP8 was co-expressed with increasing amount of Myc-SMYD3 in 293T cells. FKBP8 methylation was examined with GST-pulldown (PD) followed by WB (WCE for whole cell extract).
d. GST-FKBP8 fragments were co-expressed with Myc-SMYD3 in 293T cells. FKBP8 methylation was examined with GST-pulldown (PD) followed by WB (WCE for whole cell extract).
e. MS-MS spectrum of FKBP8 peptide containing methylated K377.
f. GST-FKBP8-WT or mutants were co-expressed with SMYD3. FKBP8 methylation was examined with GST-pulldown (PD) followed by WB.

Supplementary Figure S4 related to Figure 4.
a. FKBP8 was knocked-down in HLF cells. Cell lysates were analyzed with WB.
b. HLF KDM1A-KD and rescue cells were subject to realtime RT-PCR analysis. Error bars denote standard deviation of technical triplicates. P-values (>0.05) were calculated with one-way ANOVA followed by pair-wise comparison to the control group (*NS* denotes not significant).
c. WB for 24 pairs of HCC patient samples (N denotes normal tissues, T denotes HCC tumor tissue).
d-f. Boxplots for protein expression levels of KDM1A (d), BCL2 (e) or FKBP8 (f) in HCC patients cancer tissue and paired normal tissues. Density of each lane was normalized to HLF on the same blot. Protein expression is calculated as log2(density / density of GAPDH). P values (<0.05) were calculated from paired student's t-test.
g. FKBP8 was knocked-down in KDM1A-KD HLF cells and then FKBP8-WT or -K377R was rescue-expressed. Same number of cells were then seeded. Cell numbers were counted five days later. Fold of growth was normalized to that in control cells. Error bars denote standard deviation of four biological replicates. P-value (<0.05) was calculated with one-way ANOVA followed by pair-wise comparison as indicated.
h-j. FKBP8 was knocked-down in KDM1A-KD HLE (h), HepG2 (i) and Huh7 (j) cells and then FKBP8-WT or -K377R was rescue-expressed. Same number of cells were then seeded. Cell proliferation over five days were measured with CCK-8. Fold of growth was normalized to that in control cells. Error bars denote standard deviation of six biological replicates. P-values (<0.05) were calculated with one-way ANOVA followed by pair-wise comparison as indicated.
k. Same amount of FLAG-CaM on agarose beads were incubated with 239T cell lysates expressing GST-FKBP8 and SMYD3. Methylation levels of GST-FKBP8 in flow-through and bound fractions were analyzed with WB.

Supplementary Figure S5 related to Figure 5.

a. GST-KDM1A fragments were co-transfected with HA-KAT8 into 293T cells. Acetylation of KDM1A fragments was then analyzed by GST-Pulldown (PD) followed by WB.
b. MS-MS spectrum for KDM1A peptide containing acetylated KDM1A-K117.
c. Alignment of amino acid sequence of KDM1A from indicated species. Labelled in red are the residues corresponding with human KDM1A-K117.

Supplementary Figure S6 related to Figure 6.
a. 293T cells were co-transfected with myc-KDM1A and HA-FKBP8. Cells were analyzed with immunofluorescence with HA-tag and Myc-tag antibodies. Scale bar=10μm.
b-d. Myc-KDM1A-WT or K117 mutants were expressed HepG2 (b), HLE (c) and Huh7 (d) cells. Cells were fractionated into cytoplasm and nuclei. Shown are results from Western Blot.
e. KAT8 was knocked-down in HepG2 cells with lentivirus-expressed shRNA. Cells were analyzed with WB.
f. HLF KAT8-KD cells were analyzed with realtime RT-PCR. Error bars denote standard deviation of technical triplicates. P-value (>0.05) was calculated from one-way ANOVA followed by pair-wise comparison to the control group (*NS* denotes not significant).
g. 293T cells were transfected with myc-JADE2 and analyzed by immunofluorescence with Myc-tag antibody. Shown are photos taken with fluorescence microscopy. Scale bar=10μm.
h. Myc-JADE2 was expressed in HLF cell with lentivirus. Cells were analyzed by immunofluorescence with Myc-tag antibody. Shown are photos taken with fluorescence microscopy. Scale bar=20μm.
i. JADE2 was knocked-down in 293T cells with lentivirus-expressed shRNA. Cells were analyzed with WB.

Supplementary Figure S7 related to Figure 7.
a. Methylated GST-FKBP8 (110-389) was purified from 293T cells co-transfected with myc-SMYD3. GST-FKBP8 was then incubated with FLAG-KDM1A in vitro for demethylation. Reactant was analyzed with WB.
b. GST-FKBP8, myc-SMYD3 and myc-KDM1A were co-transfected into 293T cells. Cells were analyzed with GST-Pulldown followed by WB.
c. HLF control or KDM1A-KD cells were treated with 5μM sorafenib for 80 hours. Cells were counted before and after treatment. Fold of proliferation was normalized to that of control cells. Error bars denote standard deviation of four biological replicates. P-values (<0.05) were calculated from one-way ANOVA followed by pair-wise comparison as indicated.
d. KDM1A-WT or -K117R was rescue-expressed in HLF KDM1A-KD cells. Cells were treated with 5 μM sorafenib for 80 hours. Cells were counted before and after treatment. Fold of cell proliferation was normalized to that of control cells. Error bars denote standard deviation of four biological replicates. P-values (<0.05) were calculated from one-way ANOVA followed by pair-wise comparison as indicated.
e. KDM1A was knocked-down in sorafenib-resistant HLF cells. Cells were treated with 5 μM sorafenib for 80 hours. Cells were treated with 5 μM sorafenib for 80 hours. Fold of cell proliferation was normalized to that of control. Error bars denote standard deviation of four biological replicates. P-values (<0.05) were calculated from one-way ANOVA followed by pair-wise comparison as indicated.

f. Primary HCC cell culture 2 was treated with 10 μM GSK2879552 (GSK) and 10 μM ORY1001 (ORY) for seven days. Cells lysates were analyzed with WB.
g. Primary HCC cell culture 2 was infected with lentivirus expressing KAT8-shRNA. After selection with puromycin for five days, cell lysates were analyzed with WB.
h-i. Primary HCC cell culture 1 and 2 were treated with 5 μM sorafenib and/or 10 μM ORY1001. Cells were treated with 5 μM sorafenib for 80 hours. Fold of cell proliferation was normalized to that of control. Error bars denote standard deviation of four biological replicates. P-values (<0.05) were calculated with one-way ANOVA followed by pair-wise comparison as indicated.

Figure.S01

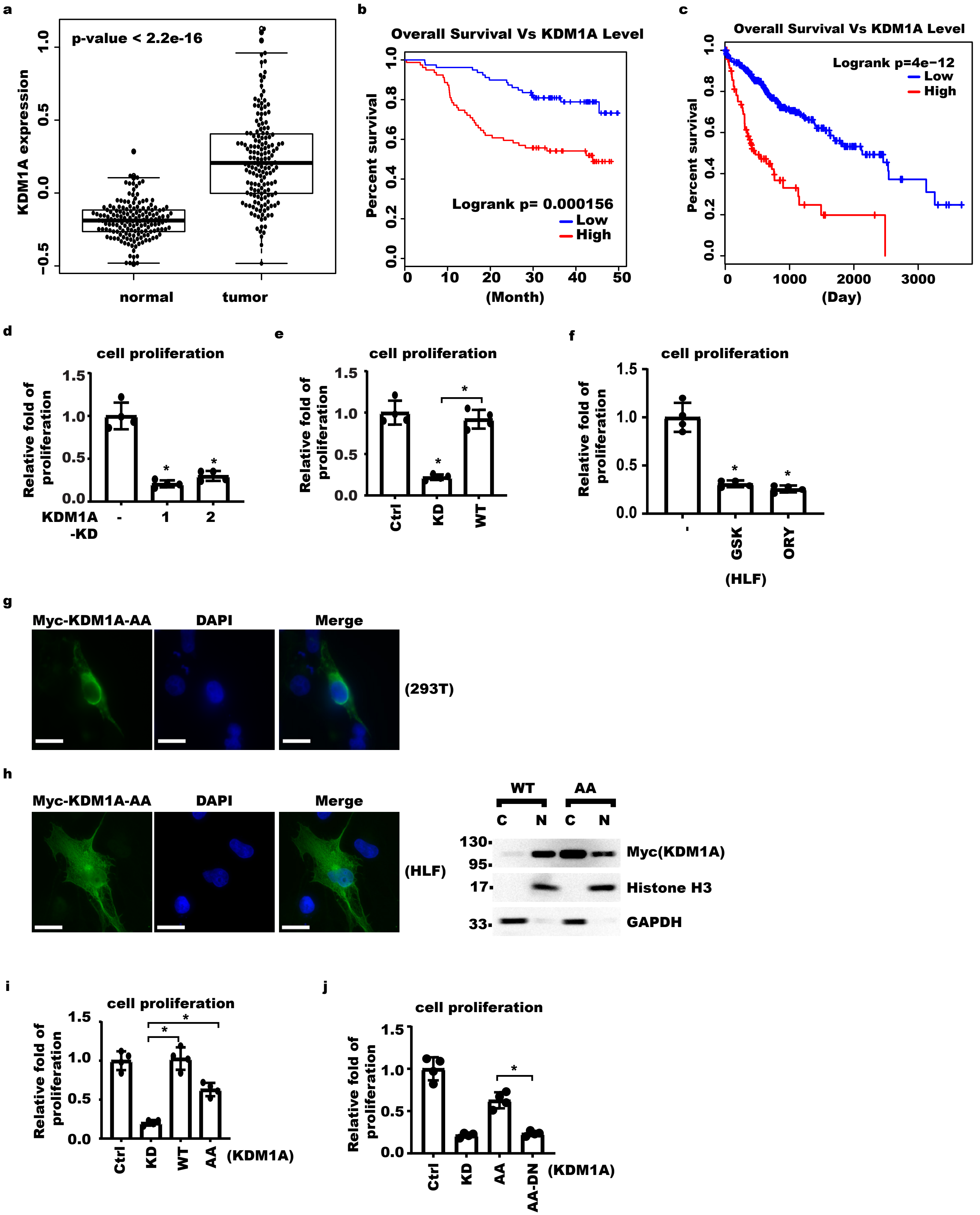

a
p-value < 2.2e-16
KDM1A expression
normal
tumor
b
Overall Survival Vs KDM1A Level
Percent survival
Logrank p= 0.000156
Low
High
(Month)
c
Overall Survival Vs KDM1A Level
Logrank p=4e−12
Low
High
Percent survival
(Day)
d
cell proliferation
Relative fold of proliferation
KDM1A -KD
e
cell proliferation
Relative fold of proliferation
Ctrl
KD
WT
f
cell proliferation
Relative fold of proliferation
GSK
ORY
(HLF)
g
Myc-KDM1A-AA
DAPI
Merge
(293T)
h
Myc-KDM1A-AA
DAPI
Merge
(HLF)
WT
AA
C
N
C
N
Myc(KDM1A)
Histone H3
GAPDH
i
cell proliferation
Relative fold of proliferation
Ctrl
KD
WT
AA
(KDM1A)
j
cell proliferation
Relative fold of proliferation
Ctrl
KD
AA
AA-DN
(KDM1A)

Figure.S02

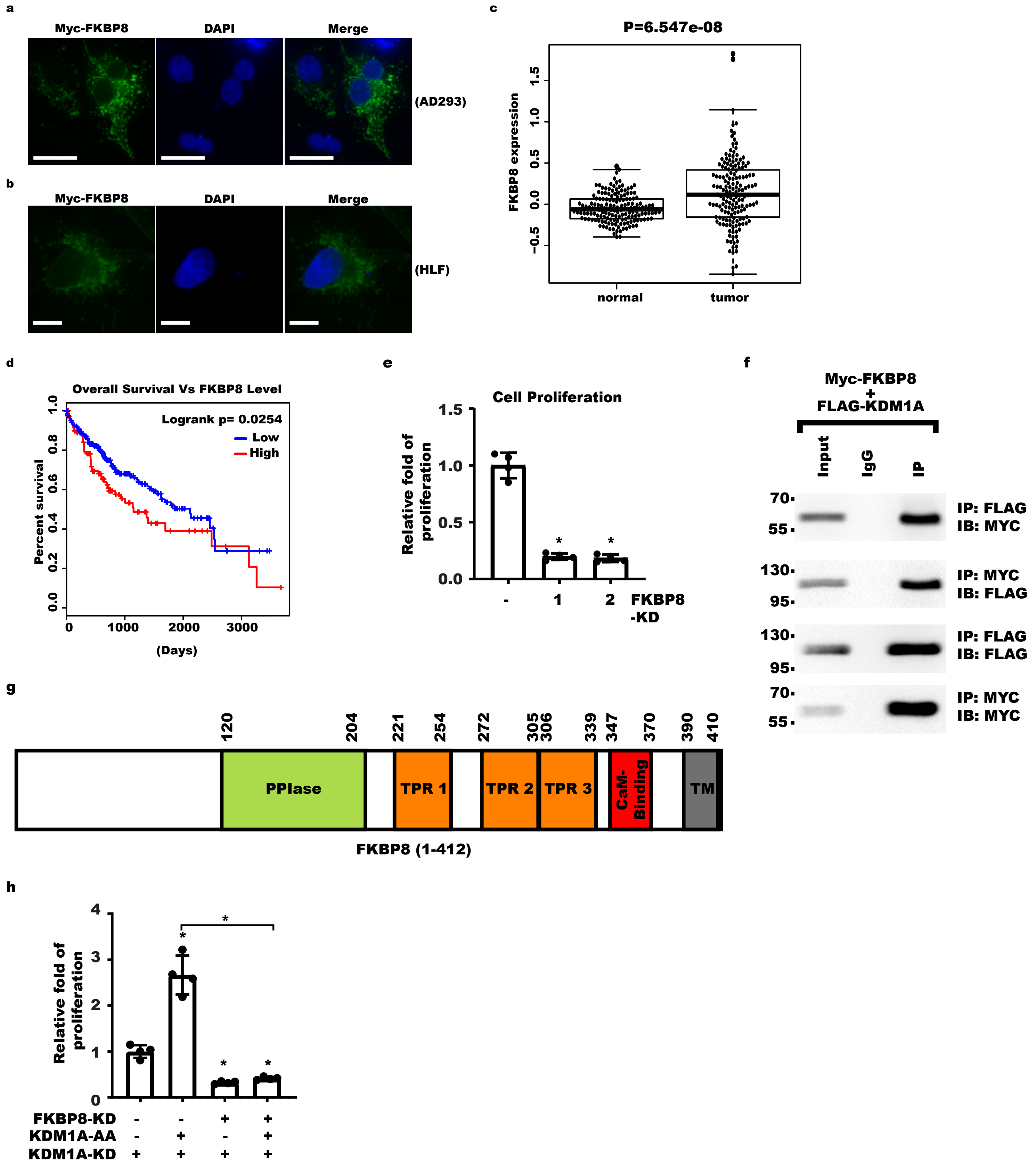
a
Myc-FKBP8
DAPI
Merge
(AD293)
b
Myc-FKBP8
DAPI
Merge
(HLF)
c
P=6.547e-08
FKBP8 expression
normal
tumor
d
Overall Survival Vs FKBP8 Level
Logrank p= 0.0254
Low
High
Percent survival
(Days)
e
Cell Proliferation
Relative fold of proliferation
FKBP8 -KD
f
Myc-FKBP8 + FLAG-KDM1A
Input
IgG
IP
IP: FLAG
IB: MYC
IP: MYC
IB: FLAG
IP: FLAG
IB: FLAG
IP: MYC
IB: MYC
g
PPIase
TPR 1
TPR 2
TPR 3
CaM-Binding
TM
FKBP8 (1-412)
h
Relative fold of proliferation
FKBP8-KD
KDM1A-AA
KDM1A-KD

Figure.S03

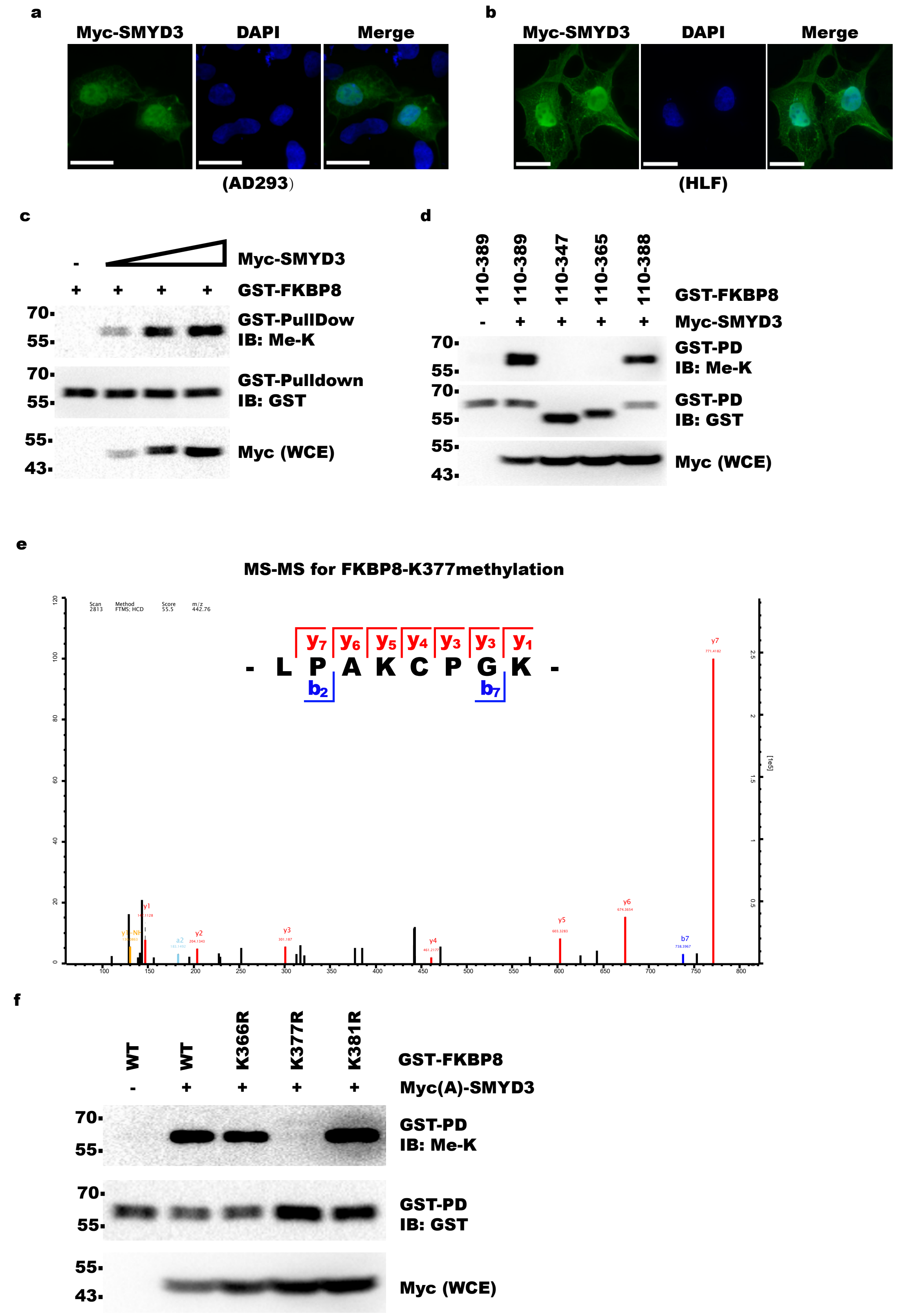

a
Myc-SMYD3
DAPI
Merge
(AD293)
b
Myc-SMYD3
DAPI
Merge
(HLF)
c
Myc-SMYD3
GST-FKBP8
GST-PullDow
IB: Me-K
GST-Pulldown
IB: GST
Myc (WCE)
70
55
70
55
55
43
d
110-389
110-389
110-347
110-365
110-388
GST-FKBP8
Myc-SMYD3
GST-PD
IB: Me-K
GST-PD
IB: GST
Myc (WCE)
70
55
70
55
55
43
e
MS-MS for FKBP8-K377methylation
Scan 2813
Method FTMS; HCD
Score 55.5
m/z 442.76
y7 y6 y5 y4 y3 y3 y1
- L P A K C P G K -
b2 b7
y1
y2
y3
y4
y5
y6
y7
a2
b7
f
WT
WT
K366R
K377R
K381R
GST-FKBP8
Myc(A)-SMYD3
GST-PD
IB: Me-K
GST-PD
IB: GST
Myc (WCE)
70
55
70
55
55
43

Figure.S04

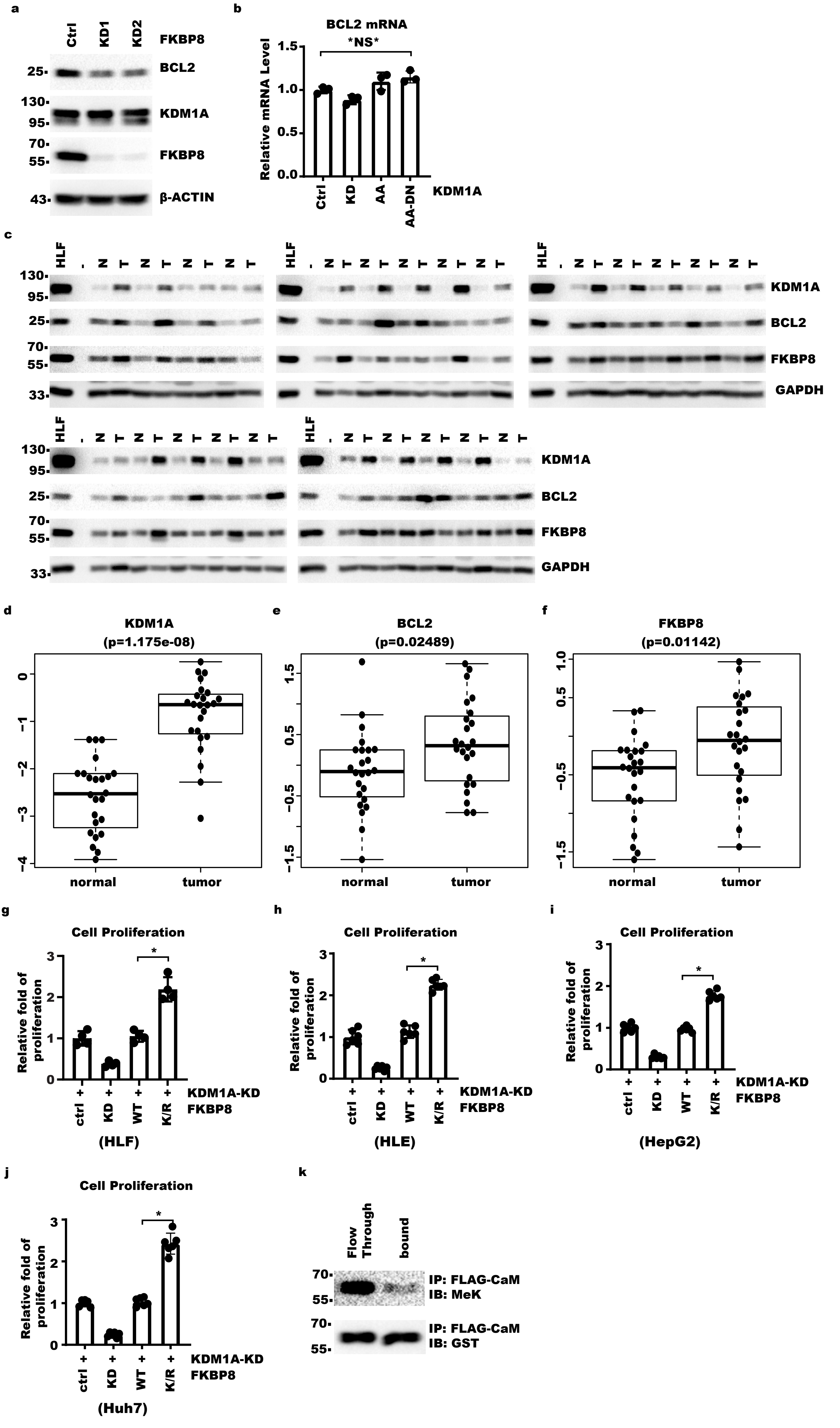
a
Ctrl
KD1
KD2
FKBP8
BCL2
KDM1A
FKBP8
β-ACTIN
25
130
95
70
55
43
b
BCL2 mRNA
NS
Relative mRNA Level
1.5
1.0
0.5
0.0
Ctrl
KD
AA
AA-DN
KDM1A
c
HLF
N
T
KDM1A
BCL2
FKBP8
GAPDH
130
95
25
70
55
33
d
KDM1A
(p=1.175e-08)
e
BCL2
(p=0.02489)
f
FKBP8
(p=0.01142)
normal
tumor
g
Cell Proliferation
Relative fold of proliferation
ctrl
KD
WT
K/R
KDM1A-KD
FKBP8
(HLF)
h
Cell Proliferation
(HLE)
i
Cell Proliferation
(HepG2)
j
Cell Proliferation
(Huh7)
k
Flow Through
bound
IP: FLAG-CaM
IB: MeK
IP: FLAG-CaM
IB: GST
70
55

Figure.S05

a

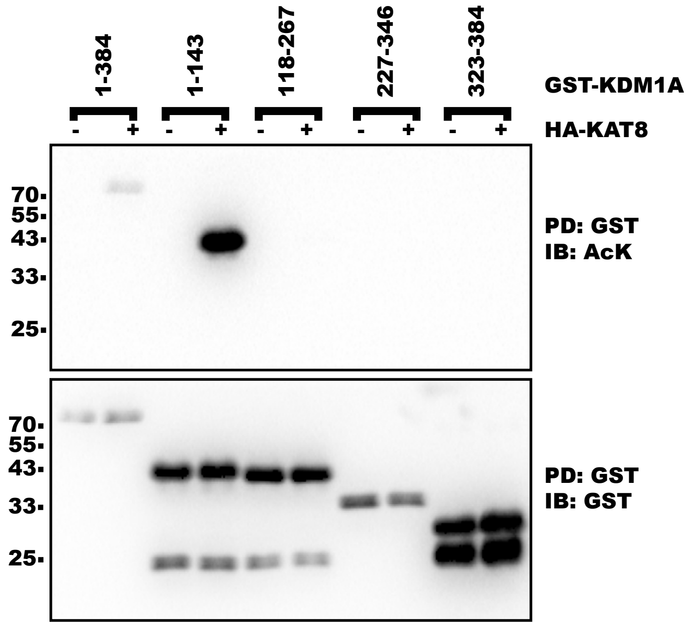

b

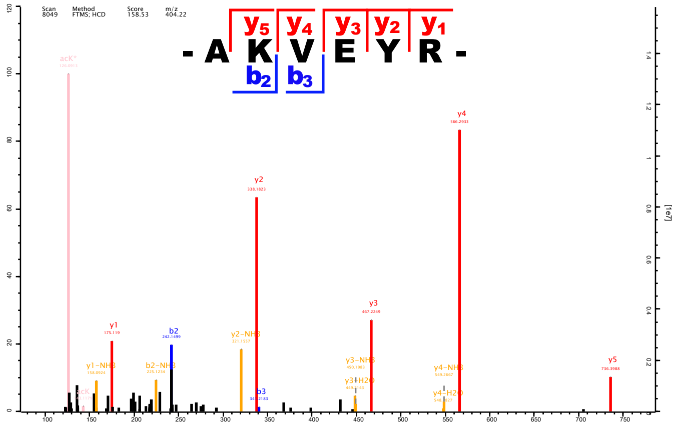

c

**Human**	**RRTSRRKRAKVEYREMDES**
**Drosophila**	**RRTSRRNRPKVDYSNRPSG**
**Zebra Fish**	**RRTSRRKRAKVEYREMDES**
**Frog**	**RRTSRRKRAKVEYREMDES**
**Chicken**	**RRTSRRKRAKVEYREMDES**
**Pig**	**RRTSRRKRAKVEYREMDES**
**Chimpanzee**	**RRTSRRKRAKVEYREMDES**
**Mouse**	**RRTSRRKRAKVEYREMDES**
**Rat**	**RRTSRRKRAKVEYREMDES**

Figure.S06

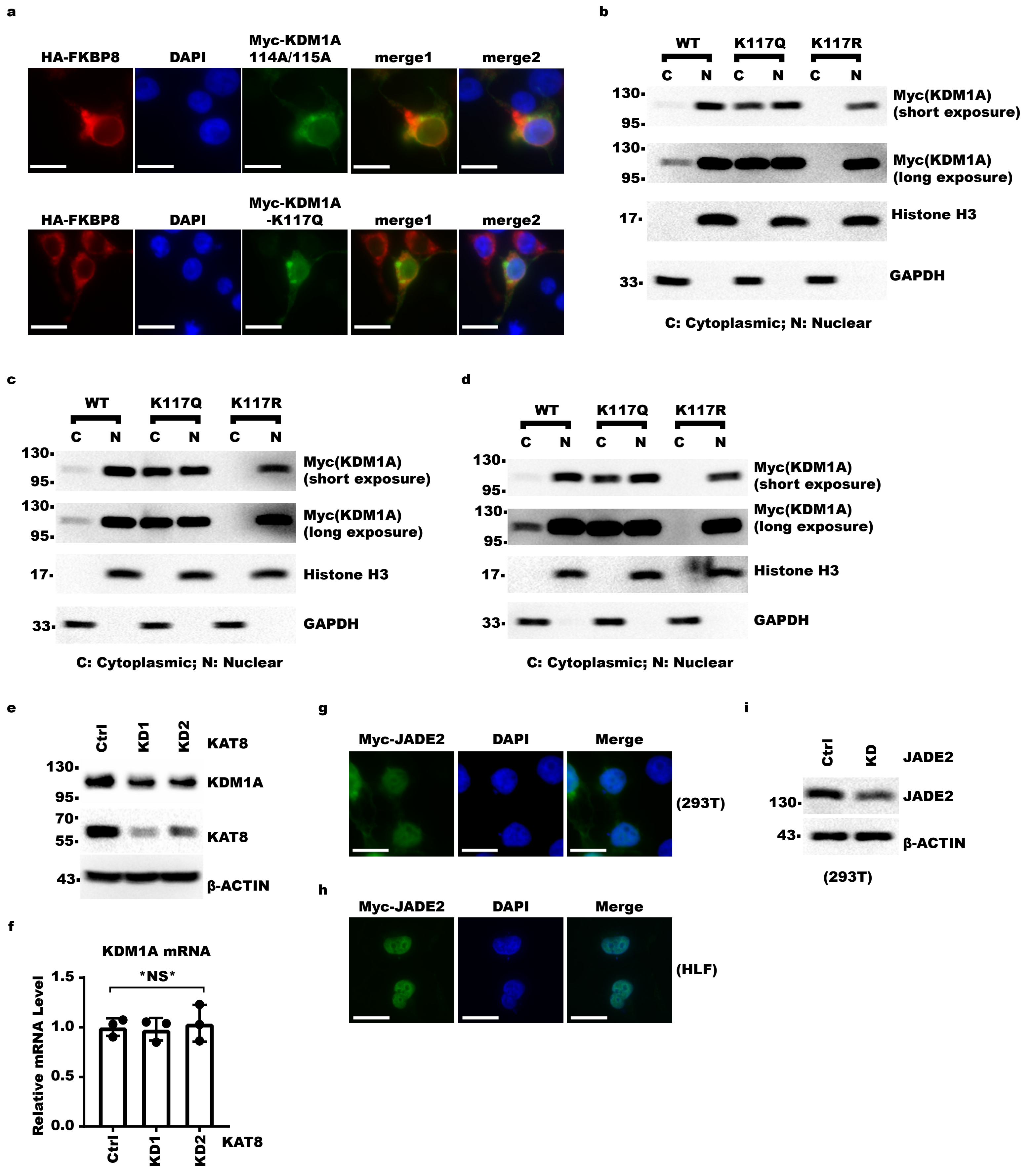
a
HA-FKBP8
DAPI
Myc-KDM1A 114A/115A
merge1
merge2
HA-FKBP8
DAPI
Myc-KDM1A -K117Q
merge1
merge2
b
WT
K117Q
K117R
C N C N C N
130
95
Myc(KDM1A) (short exposure)
Myc(KDM1A) (long exposure)
17
Histone H3
33
GAPDH
C: Cytoplasmic; N: Nuclear
c
WT
K117Q
K117R
C N C N C N
Myc(KDM1A) (short exposure)
Myc(KDM1A) (long exposure)
Histone H3
GAPDH
C: Cytoplasmic; N: Nuclear
d
WT
K117Q
K117R
C N C N C N
Myc(KDM1A) (short exposure)
Myc(KDM1A) (long exposure)
Histone H3
GAPDH
C: Cytoplasmic; N: Nuclear
e
Ctrl
KD1
KD2
KAT8
130
95
KDM1A
70
55
KAT8
43
β-ACTIN
f
KDM1A mRNA
NS
Relative mRNA Level
1.5
1.0
0.5
0.0
Ctrl
KD1
KD2
KAT8
g
Myc-JADE2
DAPI
Merge
(293T)
h
Myc-JADE2
DAPI
Merge
(HLF)
i
Ctrl
KD
JADE2
130
JADE2
43
β-ACTIN
(293T)

Figure.S07

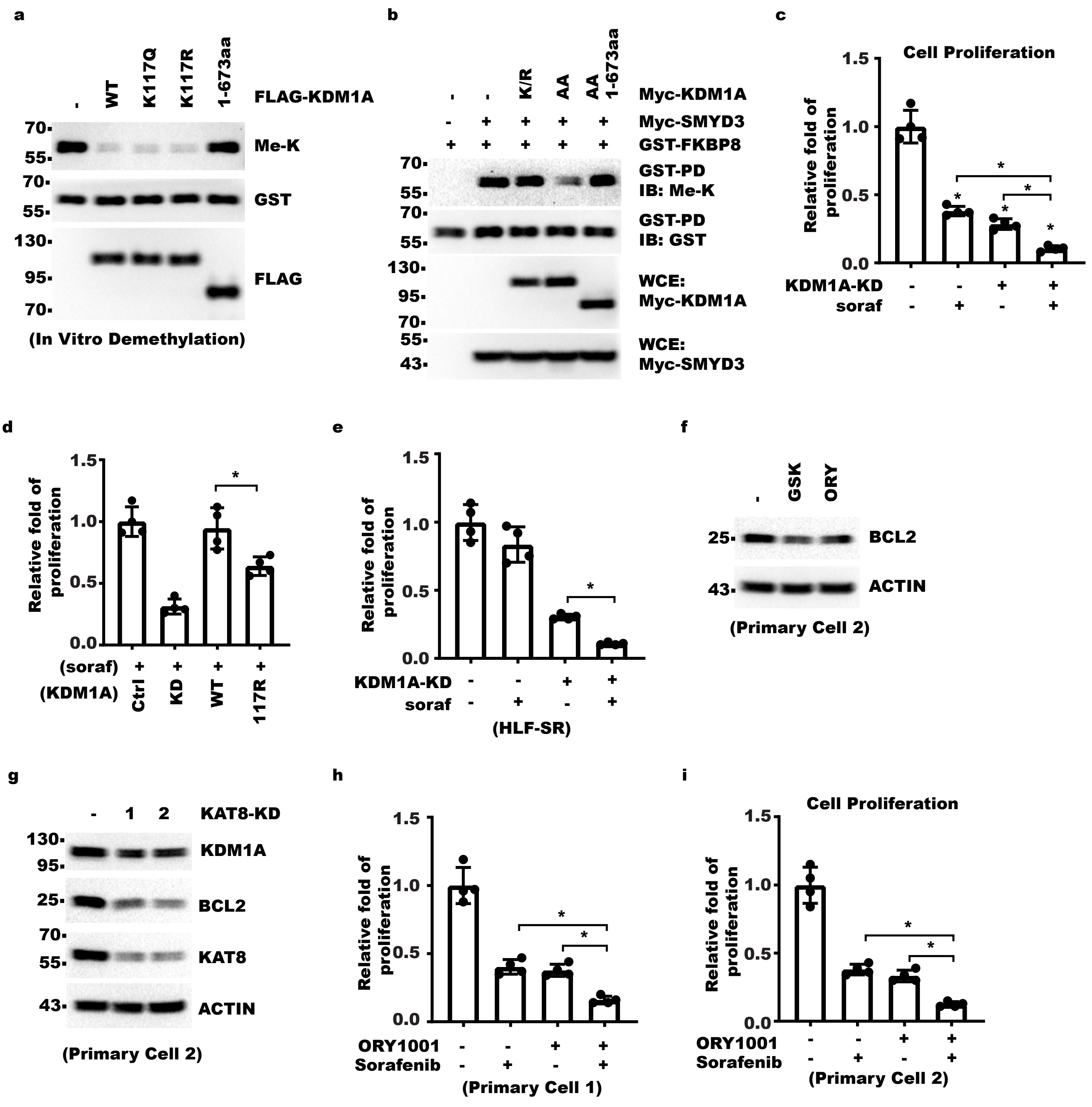

a
-
WT
K117Q
K117R
1-673aa
FLAG-KDM1A
70
55
Me-K
70
55
GST
130
95
70
FLAG
(In Vitro Demethylation)
b
Myc-KDM1A
- - K/R AA AA 1-673aa
Myc-SMYD3
- + + + +
GST-FKBP8
+ + + + +
70
55
GST-PD
IB: Me-K
70
55
GST-PD
IB: GST
130
95
70
WCE:
Myc-KDM1A
55
43
WCE:
Myc-SMYD3
c
Cell Proliferation
Relative fold of proliferation
1.5
1.0
0.5
0.0
*
KDM1A-KD - - + +
soraf - + - +
d
Relative fold of proliferation
1.5
1.0
0.5
0.0
*
(soraf) + + + +
(KDM1A) Ctrl KD WT 117R
e
Relative fold of proliferation
1.5
1.0
0.5
0.0
*
KDM1A-KD - - + +
soraf - + - +
(HLF-SR)
f
-
GSK
ORY
25
BCL2
43
ACTIN
(Primary Cell 2)
g
- 1 2 KAT8-KD
130
95
KDM1A
25
BCL2
70
55
KAT8
43
ACTIN
(Primary Cell 2)
h
Relative fold of proliferation
1.5
1.0
0.5
0.0
*
ORY1001 - - + +
Sorafenib - + - +
(Primary Cell 1)
i
Cell Proliferation
Relative fold of proliferation
1.5
1.0
0.5
0.0
*
ORY1001 - - + +
Sorafenib - + - +
(Primary Cell 2)
